# From remote sensing and machine learning to the history of the Silk Road: large scale material identification on wall paintings

**DOI:** 10.1038/s41598-020-76457-9

**Published:** 2020-11-09

**Authors:** Sotiria Kogou, Golnaz Shahtahmassebi, Andrei Lucian, Haida Liang, Biwen Shui, Wenyuan Zhang, Bomin Su, Sam van Schaik

**Affiliations:** 1 School of Science and Technology, Nottingham Trent University, Nottingham, NG11 8NS UK; 2 Dunhuang Research Academy, Jiuquan, Gansu Province China; 3 The British Library, 96 Euston Road, London, NW1 2DB UK

**Keywords:** Techniques and instrumentation, Imaging and sensing, Optical spectroscopy, Imaging studies

## Abstract

Automatic remote reflectance spectral imaging of large painted areas in high resolution, from distances of tens of meters, has made the imaging of entire architectural interior feasible. However, it has significantly increased the volume of data. Here we present a machine learning based method to automatically detect ‘hidden’ writings and map material variations. Clustering of reflectance spectra allowed materials at inaccessible heights to be properly identified by performing non-invasive analysis on regions in the same cluster at accessible heights using a range of complementary spectroscopic techniques. The world heritage site of the Mogao caves, along the ancient Silk Road, consists of 492 richly painted Buddhist cave temples dating from the fourth to fourteenth century. Cave 465 at the northern end of the site is unique in its Indo-Tibetan tantric Buddhist style, and like many other caves, the date of its construction is still under debate. This study demonstrates the powers of an interdisciplinary approach that combines material identification, palaeographic analysis of the revealed Sanskrit writings and archaeological evidence for the dating of the cave temple paintings, narrowing it down to the late twelfth century to thirteenth century.

## Introduction

**Figure 1:**
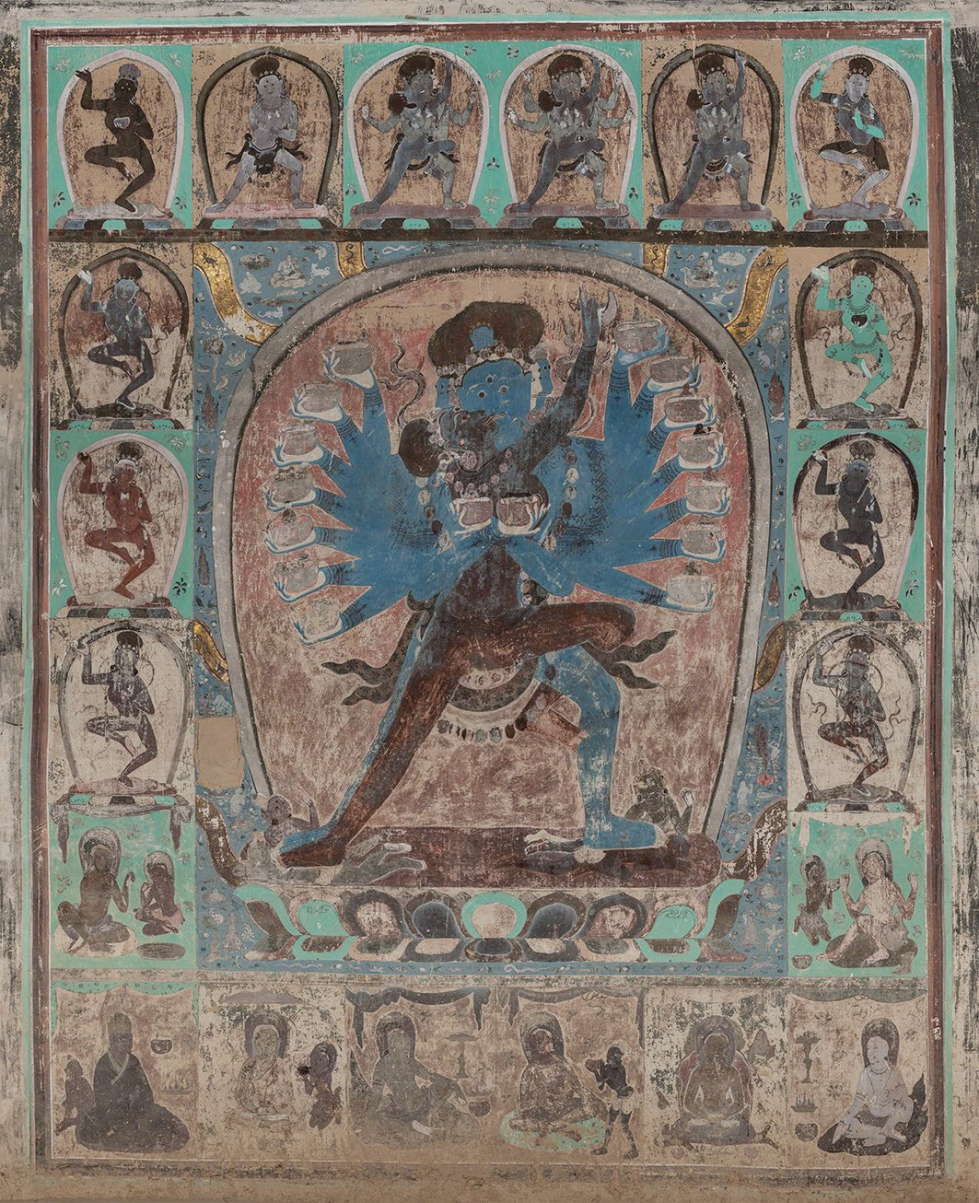
Wall painting from the main hall of Mogao Cave 465, showing the middle panel on the northern wall which depicts the multi-armed Hevajra embracing his consort and trampling on two figures; just outside the halo are scenes from the eight cemeteries with eight golden streams; these are surrounded by 12 small panels of deities in the upper parts and 8 small panels of the Great Adepts (8 of the 84) in the lower parts.

The Mogao caves in Dunhuang, at the edge of the Gobi Desert, consisting of 492 painted Buddhist cave temples dating from the fourth to the fourteenth century with 45,000 m^2^ of wall paintings, is an immense resource for the study of the history of art and architecture, religion, science and technology, politics and cultural exchange along the Silk Road. Cave 465, at the northern end of the site, is unique in its Tibetan tantric Buddhist style. Its date of construction is still under debate amongst historians and archaeologists. Its stylistic uniqueness is remarkable not only at the Mogao site but also amongst the other cave temple complexes in the Dunhuang region. The painting style is Indo-Tibetan, and it is the only one with the full range of Mahāyoga tantric Buddhist imagery, depicting wrathful deities with multiple arms and heads and in union with their consorts (Fig. 1)^1^. Historic inscriptions and graffiti were found in Chinese, Tibetan, Mongolian, ancient Uyghur, Tangut, and Sanskrit. The Dunhuang region was historically controlled by various empires such as the Chinese, Tibetan, Tangut and Mongol empires and was multi-cultural and multi-lingual with various ethnic groups, including the Uyghur, Tibetan, Tangut, Chinese and Mongolians, living at this crossroad of the eastern Silk Road. The stylistic uniqueness of Cave 465 wall paintings makes it especially difficult to date by painting style comparisons alone. Historians and archaeologists have argued for at least half a century in regard to the date of the paintings and the construction of Cave 465, with suggestions ranging from the ninth century during the Tibetan occupation^2^, the Tangut period in the eleventh–thirteenth century^3^, to the Mongol/Yuan period during the thirteenth/fourteenth century^1,4,5^.

It is important to make a distinction between the construction date of the caves and the date of the paintings. It is common for the original wall paintings in a cave to be painted over or restored in later centuries. Archaeological evidence may give indications of the date of construction and period of use of the cave temples, through a study of the excavated materials and the architectural structure of the caves. Art historical studies based on stylistic comparisons can only date the wall paintings we see today. A detailed study of the painting materials on the current wall paintings can add evidence to the date of paintings in Cave 465, since the materials, and more importantly how the pigments were used in combination, as well as the painting techniques can be indicative of a historical period. The geopolitics of a period determines the accessibility of trade routes and therefore the likelihood of technological and cultural exchanges, communication and trade.

Scientific analysis has been applied to art historical study of paintings and archaeological materials since at least the nineteenth century. Until recently, scientific analysis used to identify painting materials, such as pigments and binding media, were restricted to isolated tiny areas on artworks using either analysis of physically removed samples or non-invasive point-based analysis. Such analyses are time consuming and unrepresentative of large paintings. Recent advances in detector technology has turned various spectroscopic techniques that collect a single spectrum at a time into more efficient imaging spectroscopy techniques that collect millions of spectra simultaneously. Increasing awareness of conservation ethics is also limiting the analysis to mainly non-invasive techniques. This is also one of the reasons why ^14^C dating could not be applied in this case.

**Figure 2:**
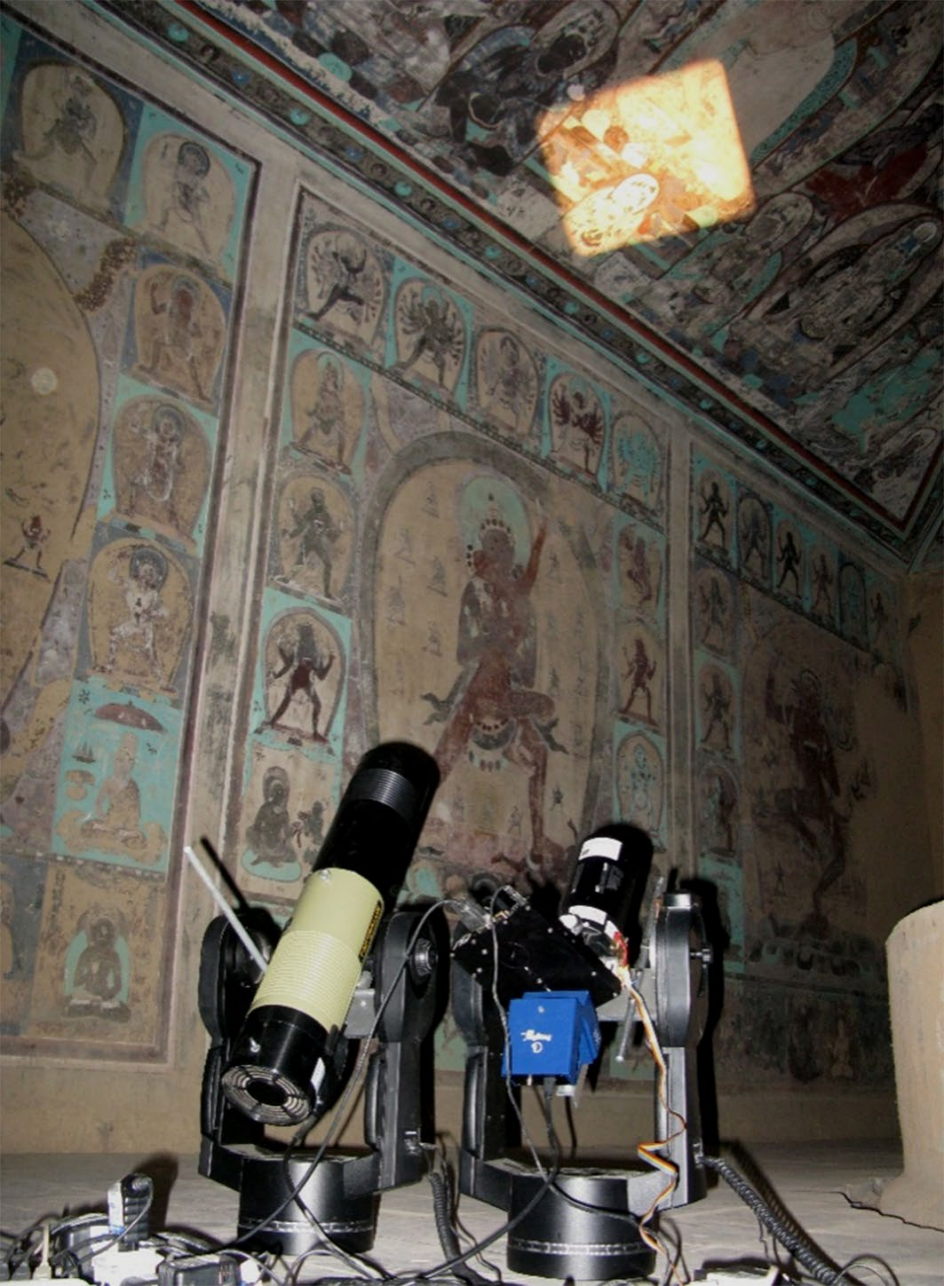
PRISMS system for automated all-cave remote spectral imaging in Mogao Cave 465.

Non-invasive reflectance spectral imaging or imaging spectroscopy in the visible and near infrared applied to paintings provides a wealth of information, including the detection of preparatory sketches, the revealing of faded writings and drawings, as well as the identification of painting materials based on their spectral signatures in the visible and the near infrared^6^. The in-house developed automated remote spectral imaging system, PRISMS, with 10 spectral bands from 400–880 nm, imaging at high spatial resolution (80 µrad) from distances of tens of meters^7^, makes it feasible to collect, from the ground, spectral images from an entire wall and ceiling paintings (Fig. 2). Spectral imaging of large areas at high resolution implies large numbers of image cubes, presenting new challenges to the data analysis.

The current study presents the development of a novel clustering algorithm using Kohonen Self-Organizing Map (SOM) and its application on the analysis of large spectral imaging datasets collected from cave temples at Mogao. Clustering methods have been widely applied for dimensionality reduction of spectral imaging data in various fields, ranging from remote sensing^8–10^ and astronomy^11^ to medicine^12^. However, automatic clustering methods on large number of image cubes have not been reported so far. Applications of clustering methods on spectral imaging data collected from paintings have been illustrated on a limited number of paintings for the identification of pigments^13–16^, binding media^17^ and substrates^18^. These methods use the ‘Spectral Hourglass Wizard’ in ENVI^19^. The drawback of these protocols is that they require the intervention of an operator in several stages of the procedure including manual selection of clusters^15^, making their application on the automatic processing of large datasets impractical. These methods may also lead to inexact spectral classification. For example, the ‘spectral angle mapper’ algorithm within ENVI is only sensitive to the spectral shape but not the intensity of the reflectance spectra. This is convenient in the field of remote sensing, as the illumination conditions are not controlled and therefore observed spectral intensity differences can be related to lighting variations, which is unrelated to the material information. However, in spectral imaging of cultural heritage objects, the illumination conditions are usually controlled and remain stable during the imaging procedure. Therefore, any observed difference in the spectral intensity can be attributed to variations in the paint mixtures, for example, different proportions of white or black paint, or variations in pigment particle size. Methods that consider only the relative reflectance can result in incomplete classification of materials. Salerno et al.^20^ suggested the application of an unsupervised method, the Kohonen Self-Organising Map (SOM)^21–23^, to cluster an easel painting based on a colour (RGB) image and a near infrared (NIR) image. However, this method was not designed to process a large number of spectral image cubes. In addition, the clustering was performed on a set of images collected in four broad spectral bands (i.e. R, G, B and NIR), which provides less accurate distinction between the various pigments compared with 10 bands.

The present algorithm is designed to overcome the limitations of the existing methods, so that large reflectance spectral imaging datasets collected from large scale paintings can be automatically clustered to form a materials cluster map^24^. SOM is chosen because it is suited to the processing of large datasets with high levels of spectral variations. It is an unsupervised technique which does not require user input and naturally reduces the spectral image cubes into a 2D map of the clusters which can be easily visualized as a materials cluster map. A reference database of unique spectra is produced from the unsupervised SOM clustering of an initial image cube, which is then used for the sequential and supervised SOM clustering of the rest of the image cubes. The reference database is constantly updated by adding clusters whenever the spectral information of a pixel does not belong to any of the existing clusters. It allows effective clustering of pixels of similar spectral reflectance (i.e. both shape and intensity) and therefore unique material composition into a manageable number of clusters. Since the processing is applied sequentially on individual image cubes, it is therefore scalable to datasets consisting of any number of image cubes. Detailed analysis to confirm the material identification can then be conducted on each of these clustered regions using a suite of complementary non-invasive spectroscopic techniques including Raman, X-ray fluorescence (XRF) and high spectral resolution fibre optic reflectance spectroscopy (FORS) from UV to short wave infrared (350–2500 nm).

Uncovered or enhanced faded or ‘hidden’ writings and preparatory sketches are often used for attribution and dating by historians. Traditionally, faded writings and preparatory sketches can be revealed in the near infrared or UV bands of spectral images. However, it was found that there were cases where these bands do not reveal faded writing, but manipulation of the entire spectral image cube revealed the writing, e.g. through weighted difference images of spectral bands chosen with a priori knowledge^7^. Rather than relying on ad hoc educated guesses, it is necessary to find an automated method to systematically detect such ‘hidden’ information when dealing with a large dataset. Principal Component Analysis (PCA) and Independent Component Analysis (ICA) have been applied to the enhancement of writings in documents with varying degrees of success^20,25–28^. A method that is based on PCA and ICA of the entire spectral cube is adopted. This method was found to not only enhance the drawings and writings that can be seen in some of the individual bands but also automatically reveal information that is not seen in any of the bands.

## Historical background

This section introduces a brief history of the periods in question within their cultural context. The Tibetan Empire (Seventh to ninth century) at its peak controlled parts of India, Nepal, China proper and Central Asia, and patronized Buddhism from the eighth century until its gradual collapse in the mid to late ninth century. It captured the Dunhuang region from the Chinese Tang Empire and ruled it from 787–848 CE. There are few extant Tibetan Buddhist paintings from this period, but cloth paintings in the Indian style with Tibetan inscriptions from the early ninth century, similar in style to the wall paintings of the same period in the Jokhang temple in Lhasa^29^, were found in the library cave (Cave 17) at Mogao. The wall paintings of this period in Dunhuang are mostly a continuation of the Tang style. Tibetan tantric Buddhism continued to be practiced in Dunhuang after the end of Tibetan rule, as is shown by the manuscripts from Cave 17^30^.

The Tanguts are a people with close ethnic and linguistic affinity to the Tibetans. The Tangut or Western Xia Empire (1038–1227 CE) controlled the eastern parts of the Silk Road. The Dunhuang region was controlled by the Tanguts from 1036–1227 CE. In the early days of the empire, an ambitious project was commissioned to translate Buddhist canons mainly from Chinese and Tibetan sources to the newly created Tangut script. The eleventh century happens to coincide with the revival of Buddhism in Tibet, mainly tantric Buddhism, when frequent pilgrimages to holy sites in East India brought back art from the Pala Empire (750–1199 CE). It was thought that Tibetans patronized East Indian artists during the eleventh–twelfth century^29^. Buddhism flourished in the Tangut Empire from the mid-twelfth century. Many Tibetan monks from different schools taught at the Tangut court, and one of these, from the early Karma Kagyü school, was given the title of Imperial Preceptor. As the Tangut state was located between the Chinese and the Tibetan states, they were influenced by both, in terms of Buddhism and artistic styles. This is best illustrated by the paintings excavated from the ruins of the Tangut northern border city of Khara-Khoto, where similar numbers of paintings in the Chinese and the Tibetan styles were found^31^.

In 1227, the Mongols led by Genghis Khan conquered the Tanguts, and the Dunhuang region came under the control of the Mongol princes in 1227 CE. It was then incorporated into Yuan territory in 1271 CE when Kublai Khan proclaimed the formation of the Yuan dynasty in China. The Mongols favoured Tibetan tantric Buddhism. Sakya Pandita of the Sakya school arrived from Tibet at Godan Khan’s court in 1247, and in 1264, his nephew Chogyal Pagpa was appointed State Preceptor by Kublai Khan. It is also known that after the collapse of the Tangut state, many Tangut monks continued to work in the Mongol Yuan empire^32^, spreading tantric Buddhism even to Southeast China. Tibetan paintings in the thirteenth century became more Nepalese in style, after the decline of Buddhism in India as a result of Muslim conquests of Northern India towards the end of the twelfth century. In the thirteenth century, Sakya school enlisted Nepalese artists for tantric Buddhist paintings and they brought about 80 Nepalese artists to the Yuan court in present day Beijing in 1261^29^. From the fourteenth century, a variety of schools of Tibetan painting developed, drawing on both Nepalese and Chinese styles.

Dunhuang declined towards the end of the Yuan dynasty (1271–1368 CE) when the Silk Road was gradually replaced by the maritime trade route.

## Results

### Clustering of reflectance spectra and material identification

**Figure 3:**
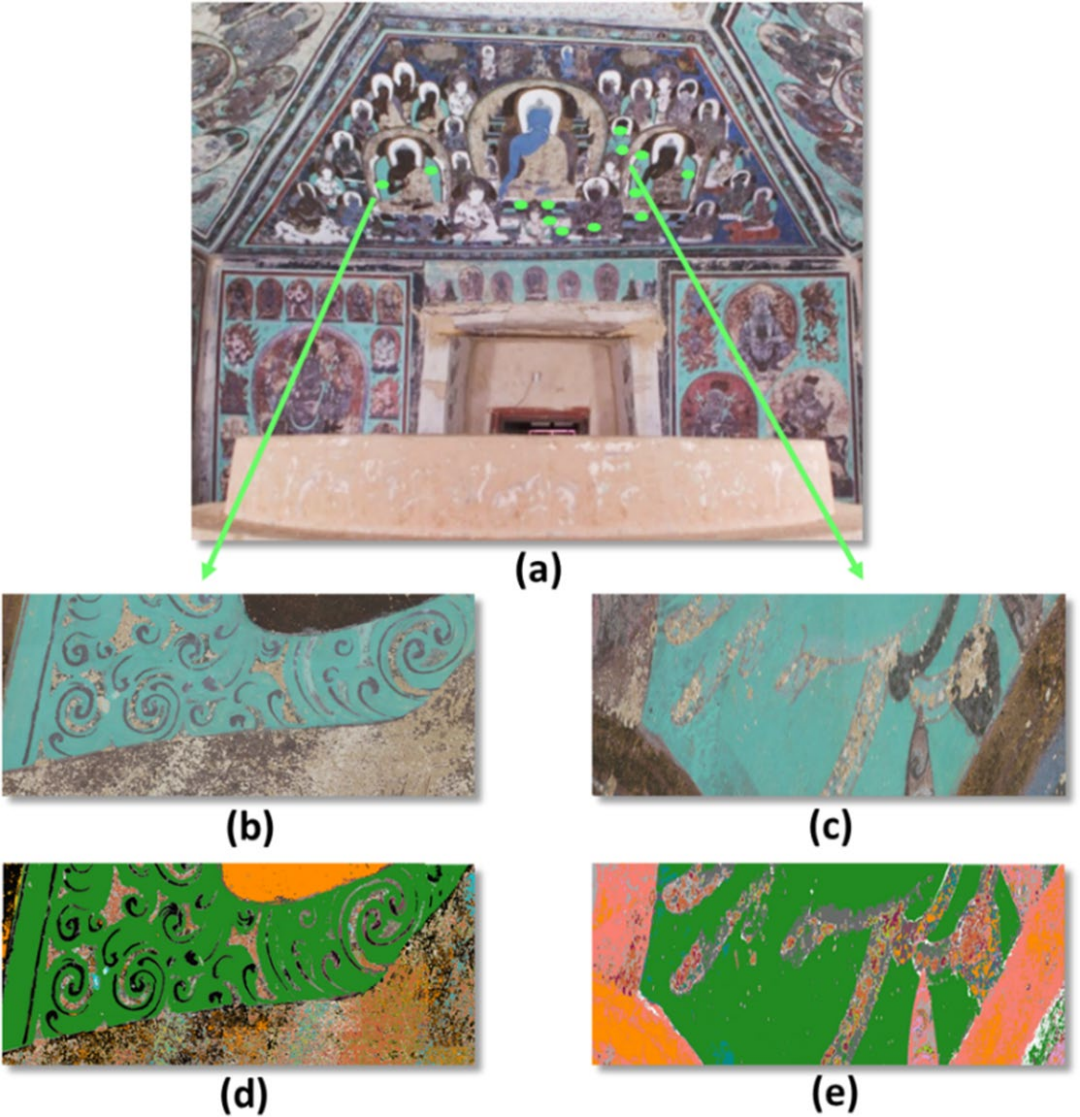
(**a**) Colour image of the east side of the main hall of Cave 465; The bright green dots on the ceiling indicate green areas in different parts of the east ceiling that share the same spectral information; (**b**,**c**) Colour images derived from the mosaic of PRISMS spectral imaging data collected from some of these areas assuming CIE illuminant D65, 1931 2° standard observer, along with (**d**,**e**) their corresponding cluster maps. Each cluster is given a unique false colour in the cluster maps.

Cave 465 originally consisted of a front hall which collapsed before the twentieth century, a middle hall (~ 7.0 m × 6.4 m and ~ 4.9 m high with slopped ceilings) with paintings of a stupa on each wall, and a main hall at the back (~ 9.9 m × 9.0 m and ~ 5.5 m high with sloped ceilings) covered with paintings from ceiling to wall. The PRISMS system was used to automatically capture large areas of wall paintings in Cave 465. For example, the eastern ceiling of Cave 465 covers an area of approximately 10 m^2^, corresponding to a total of around 5000 image cubes. Given the high spatial resolution of the PRISMS spectral image cubes, the dataset consists of over 10^9^ spectra. The exposure of the wall paintings to natural environmental conditions over the years resulted in complexity in their material composition (i.e. ranging from the original materials to those subjected to different levels of chemical degradation and physical weathering). This translates to copious spectral variations in the large dataset. After clustering, the number of unique spectra over the east ceiling was narrowed down to 960 clusters with ~ 300 of them corresponding to areas of physical damage such as cracks or exposed substrate under partially delaminated paint layers. Even though some of these clusters are not useful for a survey of material content, they are useful in a conservation survey. The idea is to capture all the information at this initial stage. Figure 3 shows that the new clustering method grouped all the areas of the ceiling that have similar spectra together, even when they are from different spectral image cubes and widely separated from each other.

**Figure 4:**
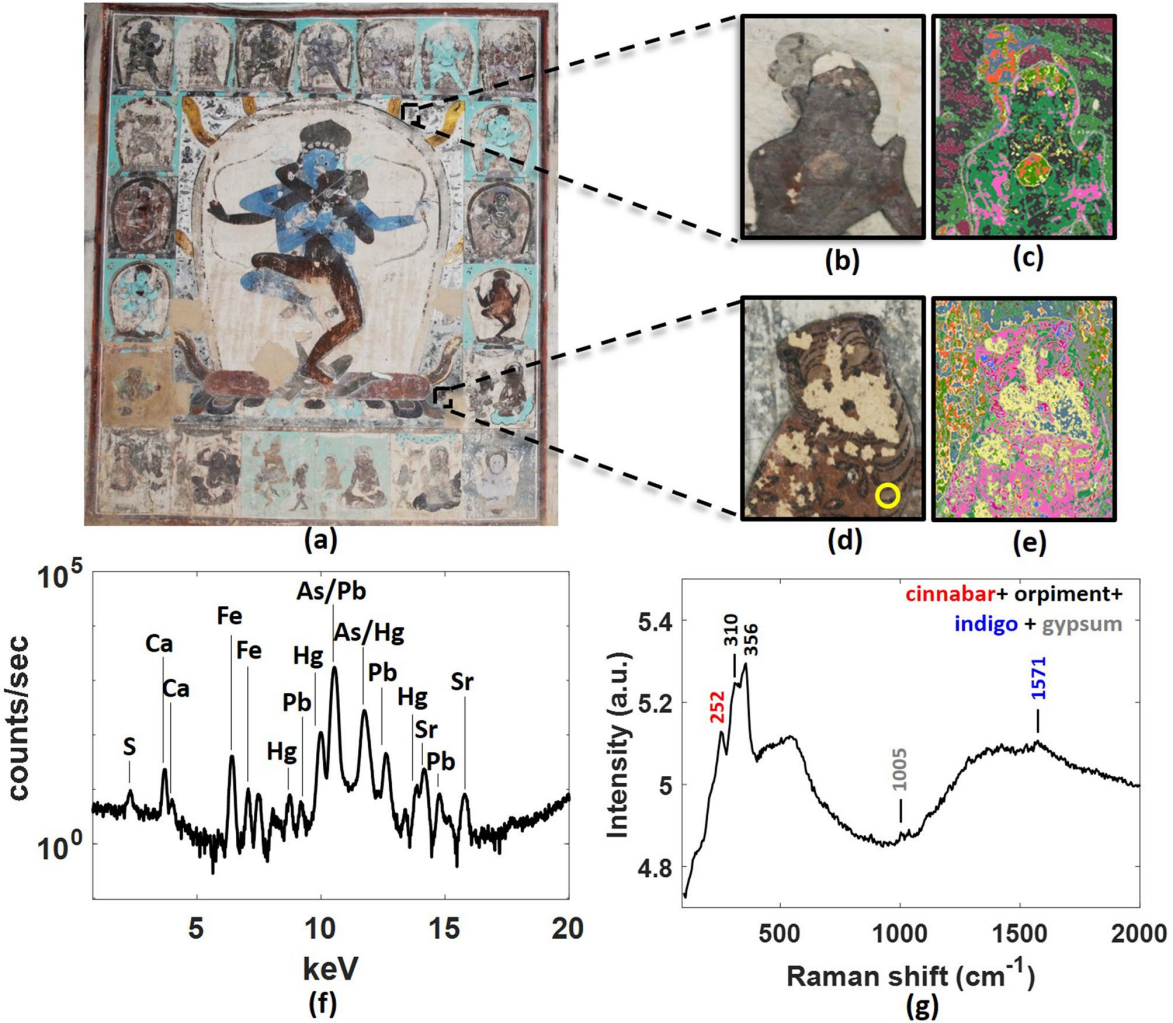
(**a**) Colour image of the eastern panel of the southern wall of the main hall of Cave 465 showing Mahāmāyā and his consort Buddhaḍākinī in union; (**b**,**d**) Two zoomed-in areas from the top and bottom of the wall along with their corresponding cluster maps (**c**,**e**) with a unique false colour assigned to each cluster; (**f**) XRF spectrum and (**g**) Raman spectrum of a region on the bottom figure (marked by a yellow circle) in one of the clusters represent by the dark green false colour in the cluster maps.

**Figure 5:**
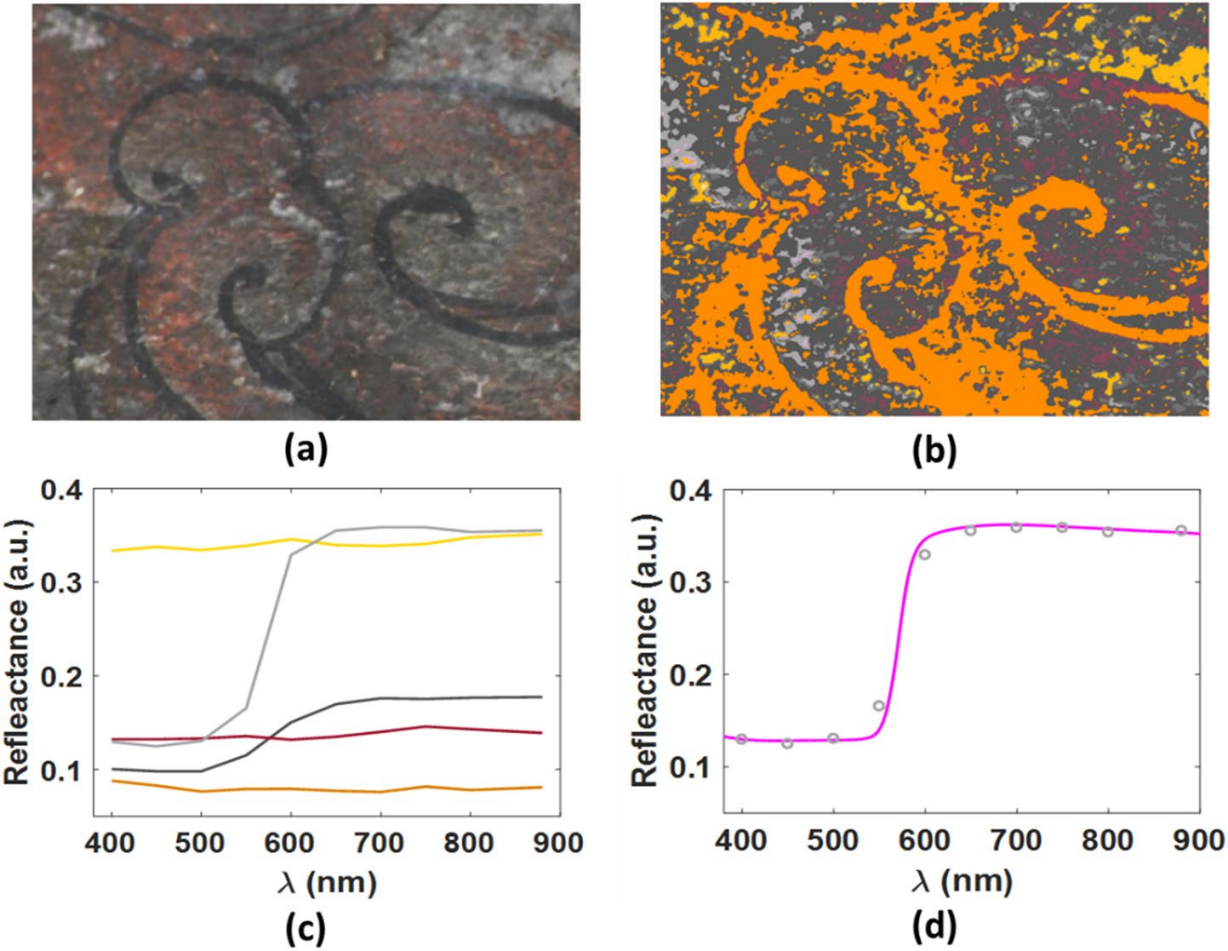
(**a**) Colour image derived from PRISMS data collected from an area of the east ceiling of Cave 465, assuming CIE illuminant D65, 1931 2° standard observer; (**b**) the corresponding cluster map with a unique false colour assigned to each cluster; (**c**) mean spectra of the various clusters describing the material variations, colour coded to match the colours of the cluster map in (**b**); (**d**) The spectrum of the cluster colour coded to light grey (light grey circles) compared with the standard spectrum of red lead (magenta curve).

While the reflectance spectrum of each cluster gives a preliminary identification of the materials, more definitive identification was achieved through the application of a suite of complementary non-invasive spectroscopic techniques such as Raman, XRF and FORS. However, these complementary techniques can only be applied locally at accessible heights near the ground. Clustering allowed these detailed identifications to be extended to regions at inaccessible heights. Figure 4 illustrates such an example at the east panel of the southern wall. As it is shown, the body of a figure at the top part of the wall was clustered with parts of the tiger figure at the ground level. The multi-modal, non-invasive analysis of the tiger figure suggested that the paint layer was a combination of cinnabar and orpiment on top of the white/blue background of indigo and possibly gypsum. The presence of scaffolds in our last field trip, allowed the performance of multi-modal complementary measurements directly on the figure at the top of the wall which confirmed that it had the same composition as the tiger figure as expected from the clustering results. The results of detailed material identification for the other clusters can be found in Supplementary Note 1 and summarised in Table 1. In addition to Table 1, black ink is often applied in a thin wash over a paint layer to achieve a darker colour (Fig. 5). Red organic dyes have not been confidently identified and therefore not included here. Apart from indigo, identification of organic dyes on these wall paintings are notoriously difficult, especially with in situ non-invasive techniques because of the ageing and loss of the organic materials over the years. The pigment combinations presented for the various coloured areas in Table 1 do not include contribution from the uniform background over which these colours are painted. A rich combination of pigments was used to achieve a desired colour. The combination of azurite and indigo to achieve a darker blue and the combination of cinnabar and orpiment to achieve an orange colour appear to be characteristic of Tibetan paintings^33–35^. Similar combinations have been found on the eleventh–fourteenth century wall paintings in Shalu (or transcribed in Chinese *pinyin* as Xialu) temple in Tibet^33^. The combination of indigo and orpiment to make a green is found in Tibetan thangkas attributed to Nepalese influence^34,35^.

**Table 1:** Pigment composition of Cave 465.

Colour	Cave 465
Red	Cinnabar Cinnabar + orpiment Cinnabar + orpiment + red lead Red lead + orpiment Red ochre
Blue	Indigo Azurite Indigo + azurite
Green	Atacamite Indigo + orpiment
White	Gypsum + dolomite
Brown/black	Cinnabar + plattnerite* Azurite + plattnerite*
Yellow	Orpiment
Lilac	Cinnabar + plattnerite*

*Degradation product of red lead (turns it from red to brown/black).

Different painting materials or combinations of pigments may have been used in different periods and from different cultural groups. Often it is the combination that gives the clue, since most mineral pigments are used across geographical and cultural boundaries. A detailed comparison between the pigment composition of Cave 465 with those of dated Mogao caves of the Tibetan, Tangut and Mongol/Yuan periods, can shed light on the date of Cave 465 wall painting. A number of the caves thought to be iconic of these three periods were selected as comparison caves and analysed using similar methods as applied to Cave 465. Cave 159 was thought to be an iconic Tibetan period temple featuring the Tibetan king; Cave 65 was thought to be an iconic Tangut temple with dated Tangut inscriptions (1085 AD); and Cave 95 was a well-known Yuan cave. In addition, the corridor of Cave 159 was renovated during the Tangut period and hence included here as a Tangut example. Similarly, Cave 386 was an early Tang temple but later renovated in the Tibetan period. Cave 97 wall paintings were catalogued as late Tangut (1140–1227 AD) by Dunhuang Academy^36^ but later changed to Uyghur^37^ which would imply early eleventh century^38^. The general uncertainty on the dates of Tangut caves at Mogao will be discussed later. Supplementary Table S1, S2, S3 summarize the pigment composition of these comparison caves for the Tibetan (eighth–ninth century), Tangut (eleventh–thirteenth century) and the Mongol/Yuan (thirteenth–fourteenth century) periods. Cave 465 has by far the most complex combination of pigments compared with the other cave temples studied here. Cinnabar, red lead or a combination of the two were used in all the caves studied. Red ochre appears to be more prevalent in the earlier period than the later periods. The blue pigment lapis lazuli from Afghanistan was used in the two Tibetan period paintings and the Tangut paintings in Cave 65, but it was not used in caves 97, 95 and 465. The combination of azurite and indigo for dark blue, characteristic of Tibetan paintings, was only found in Cave 95 and Cave 465. Talc was used in the Tibetan and Tangut paintings in caves 159 and 65 for white, while a combination of gypsum and dolomite were used in the white areas in caves 95, 97 and 465. For the greens, both malachite and atacamite were found in the Tibetan Cave 159, but only atacamite was found in the other caves except for Cave 95 where the copper containing pigment was not identified. The brown areas tend to contain plattnerite which is a degradation product of red lead and is brown/black in colour^39,40^. None of the Tangut paintings had yellows and the Tibetan Cave 159 had only small areas of yellow which were identified with yellow ochre. Both orpiment and yellow ochre were found in the Mongol/Yuan paintings in Cave 95, but the only yellow pigment found in Cave 465 was orpiment. We can separate the comparison caves into two groups in terms of affinity in material composition: (1) caves 97 and 95, and (2) caves 159, 386 and 65. The pigment composition of Cave 465 is closest to Cave 95.

To increase the number of comparison cases, earlier work conducted by the Dunhuang Academy using X-ray diffraction (XRD) and X-ray Fluorescence (XRF) spectroscopy of samples collected from cave temples, at Mogao and other neighbouring cave temple sites in the Dunhuang region within a distance of 200 km, dated to these three periods can also be included (Supplementary Note 3; Supplementary Table S4, S5, S6)^41–43^. This adds material information from 4 Mogao cave temples with paintings dated to the Tibetan period, 6 caves from the Tangut period (3 from Mogao site, 1 from Yulin site and 2 from the East Thousand Buddhas Caves) and 5 from the Mongol/Yuan period (1 from Mogao, 3 from Yulin and 1 from the East Thousand Buddha Caves). The results obtained using XRD and XRF analysis of a few physical samples per cave are not exactly comparable with the methods used in our current study where non-invasive spectral imaging of large areas has been used to form a representative view of the whole painting. While XRD can identify crystalline materials, it is not sensitive to non-crystalline substances such as most organic pigments. As an example, the XRD/XRF analysis of samples collected from wall paintings in Cave 465 found red ochre, azurite, atacamite, red lead/plattnerite and dolomite^41^ which are a subset of the pigment composition found using our current method (Table 1). Another potential caveat to consider is that while the Mogao, Yulin and East Thousand Buddha cave sites are all in the Dunhuang region, slight differences in artistic style have been noted between the sites. For example, the differences in style between the Tangut paintings at Mogao and Yulin were thought to be due to the relative political importance of Yulin over Mogao during this period^4^.

If we include the data from XRD/XRF analysis of extracted samples, we have a total of 6 comparison caves from the Tibetan period, 9 from the Tangut period and 6 from the Mongol/Yuan period. The enlarged comparison data is found to be consistent with our earlier conclusions. Talc and calcite, but never gypsum, were used for white on the Tibetan period paintings; and talc was never used on the Yuan period paintings. The pigment combination of Cave 465 suggest that it is unlikely to be from the Tibetan period. The overall pigment composition of Cave 465 is still most consistent with the Mongol/Yuan period, though Tangut period cannot be ruled out. We would have been able to give a more definitive answer based on the material analysis, if the attribution of the Tangut caves were more secure. Until 1964, it was thought that there were only a few Tangut caves at Mogao. An interdisciplinary study conducted in 1964 involving art historians, archaeologists and linguists re-classified under the Tangut period over 80 caves which were originally thought to be from the 10th to early eleventh century^44^. Cave 65 was in this category but is currently contested^44^. In the 1990s, the caves designated as late Tangut were re-assigned as Shazhou Uyghur caves, that is early eleventh century^38,45^. Cave 97 is such an example^45^. It appears that both Cave 65 and Cave 97 in Supplementary Table S2 are not securely dated to the Tangut period based on studies involving only humanities disciplines. Based on the material analysis, it would seem more consistent if Cave 65 was from an earlier period and Cave 97 was from a later period. By conducting large scale material analysis of more caves assigned to the Tangut period at the Mogao site, it will not only help with dating of Cave 465, but also help in determining which caves are from the Tangut period.

### Automatic uncovering of ‘hidden’ writing

**Figure 6:**
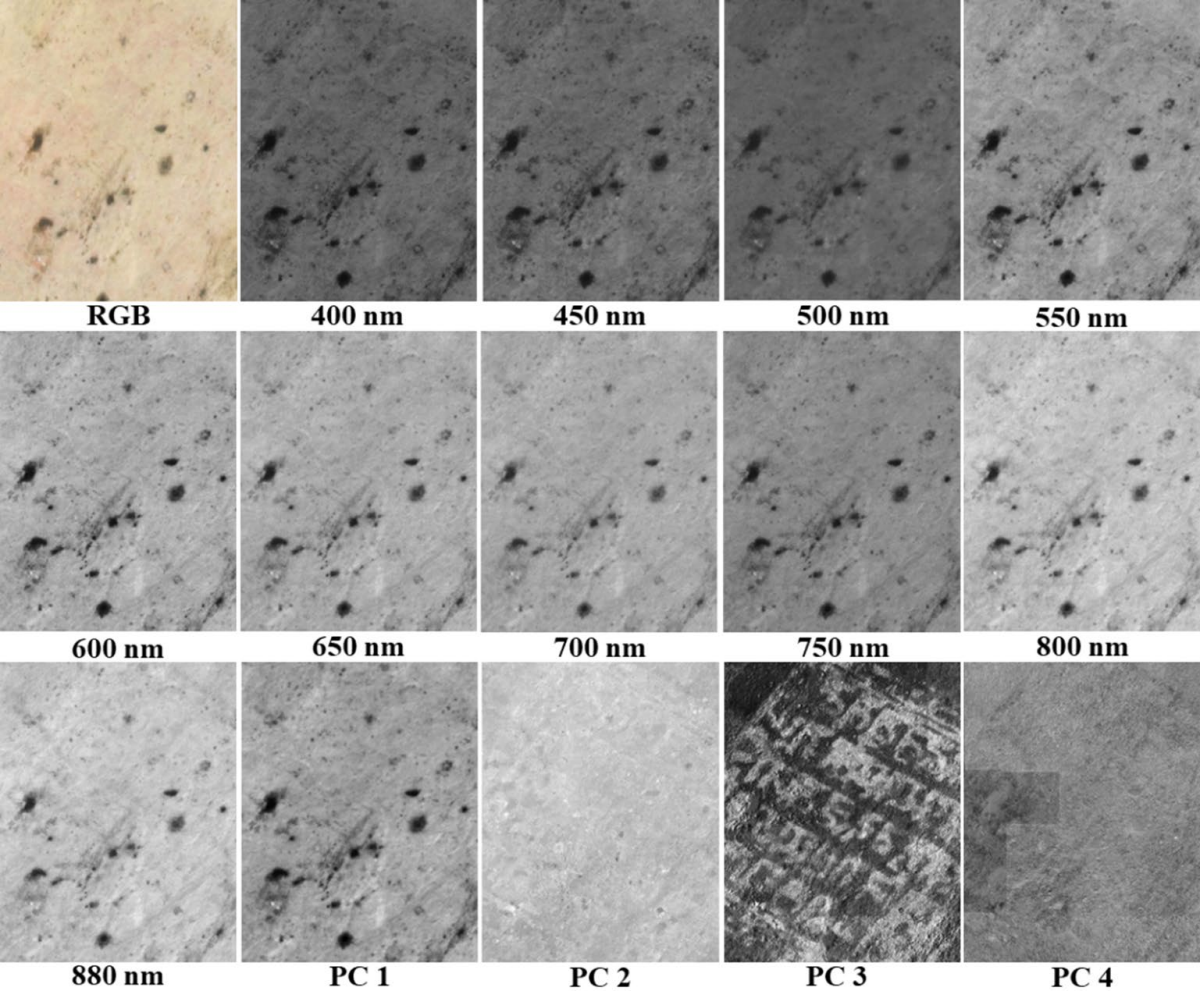
Colour image derived from PRISMS data collected from an area on the eastern ceiling with faded writings, along with the spectral images at the individual bands and the first four principal component images from results of PCA on the ten spectral bands. PC 3 reveals the faded Sanskrit writings. The colour image is derived from the spectral images assuming CIE illuminant D65, 1931 2° standard observer.

Figure 6 shows an example of PCA analysis of spectral images of faded writings on the west ceiling of Cave 465, where neither the colour image nor any of the individual spectral bands reveal any writing. The application of PCA using the correlation matrix of the spectral imaging data clearly unveiled the faded Sanskrit writings. The first principal component (PC) by definition has the largest variance and tends to give an image that is close to an average of the different spectral bands, the 2nd and 3rd PCs tend to reveal ‘hidden’ writings and drawings while the later PCs reveal imperfections in the data such as slight mismatch in intensity between neighbouring images in the final mosaiced image. This is similar to the observations made by those using PCA to enhance writings on historical manuscripts^20,25,28^. The writings were found to be printed or stamped Sanskrit text in cinnabar with decorated borders about a few centimetres square on a piece of paper which was then pasted on the ceiling. The sheet of paper on the west ceiling appears to have been glued to the ceiling face down as the letters revealed by PCA are flipped (Fig. 6). This also explains why the red writing can hardly be seen by the naked eye. ICA was also performed on this example, but the results were similar to those of PCA. In most cases, ICA gave similar results as PCA, but in some cases there were subtle differences in the clarity of the revealed writings or drawings. Consequently, the image cubes were processed by both PCA and ICA.

The ceiling is painted with the Five Celestial Buddhas, with Vairocana painted in white in the centre of the ceiling, Akṣobhya painted in blue on the east ceiling, Ratnasaṃbhava in reddish brown on the south ceiling, Amitābha in yellow on the west ceiling and Amoghasiddhi in green on the north ceiling (Supplementary Fig. S19). The Five Celestial Buddhas are identified by their hand gestures (mudrā), however, the colours of the south and west Buddhas appear to have been swapped compared to usual convention. Paper sheets with the above printed text were found at the bottom of the Buddha images on the four ceilings corresponding to the four cardinal directions. Supplementary Fig. S19 shows the best processed images of the 4 stamped text using the spectral imaging data. Closer examinations of the text and their decorative borders show that they were clearly not stamped by the same seal and the paper were of different quality and size. For example, the letters on row 2 column 5 of the 4 sheets are clearly different. The paper on the east ceiling is the thinnest and most transparent, while the paper on the north ceiling is rectangle in shape unlike the square shape of the others.

The revealed text is identified as a Buddhist Sanskrit phrase known as the “Summary of Dependent Origination". Considered a summary of the teachings of the Buddha, it can be translated as: “All things arise from causes. The Tathāgata has proclaimed these causes, as well as their cessation. This is the teaching of the Great Ascetic”, with the usual Sanskrit being: “*ye dharmā hetuprabhavā hetuṃ teṣāṃ tathāgato hy avadat teṣāṃ ca yo nirodha evaṃ vādī mahāśramaṇaḥ*”. The pasting of the paper sheet with the Sanskrit dhāraṇī accords with how this Sanskrit text is used in other contexts across the Buddhist world in the consecration of stupas, statues and painted figures^46^. Here, the paper prints seem to have been produced and pasted on the ceiling during the construction of the cave temple, as part of a consecration ritual. The Sanskrit text is written in a form of the Nāgarī script which developed in India^47^. The form of script seen in these paper prints is also seen in the Turfan manuscripts and seems to have become popular across Central and East Asia in the Tangut and Mongol empires. For example, the inscriptions carved into the rocks at Feilaifeng in Hangzhou dated to 1287–1292 AD^48^ are of a similar style. Printed texts of the same phrase in a similar script has also been found inside miniature stupas in other caves dated to the Mongol/Yuan period (e.g. caves 462 and B168) close to Cave 465 at the Mogao site^49^. Palaeographic analysis of the script (Supplementary Note 4) suggests that these were produced from after the late twelfth century when compared with a reference of Nāgarī scripts collected from dated inscriptions from all over India between ninth–thirteenth century^50^, which means the paintings could not have been consecrated in the early Tangut period. The most likely date of the ceiling paintings based on the stamped texts is, therefore, late Tangut or Yuan, consistent with the conclusions based on the painting materials composition.

## Discussion

In this section, we will consider the above results in the context of a range of interdisciplinary evidences regarding the history of Cave 465.

Those advocating the Tibetan period relied on a row of writing in Tibetan by the entrance to the main hall of the cave temple which suggested that construction of the cave temple was completed in the year 838 AD^2^. However, this date is controversial because this ‘headless’ Tibetan script became popular much later than the ninth century and the date was not written in the usual Tibetan way of naming the years (c.f. in Mogao Cave 365 the inscription of 832 and 834 AD followed the usual Tibetan convention)^51^. The Mahāyoga tantric Buddhist imagery with typical wrathful deities and scenes of deities with their consorts in sexual union depicted in Cave 465 (Fig. 1) is also surprising for ninth century paintings, since there are no extant Buddhist imageries of this kind anywhere in the Buddhist world until at least after the eleventh century despite the existence of earlier tantric texts^52^. If this is indeed from the ninth century, then it would be a highly significant discovery in Buddhist art history. The pigment analysis results from our study shows that it is highly unlikely to be of the Tibetan period.

The suggestions for the early Tangut period relied mainly on art history arguments through interpreting the iconography combined with a knowledge of the historical context and the scripture of various Tibetan tantric Buddhist sects^3,51,53^. Xie^3,51^ argued that since most of the other cave temples constructed in the Mongol/Yuan period did not have graffiti in Tangut, unlike Cave 465 which has many Tangut graffiti in the middle hall, Cave 465 must have been in existence earlier than the other Mongol/Yuan caves. It should be noted that Tangut was still in use in the Dunhuang region well after the Mongol conquest. It was also suggested that the similarity of the wall paintings in Cave 465 with thangkas excavated from Khara-Khoto points to a Tangut origin. However, it was thought that those thangkas that looked similar were originally from Tibet and dated to after 1189 CE to probably 1227 CE, but certainly before the 1380 s since that was the date of the latest securely dated painting from Khara-Khoto^31^. The painting design, that divides the painting surface into a series of rectangles with smaller rectangles all around (Fig. 1), was thought to be like those of early Tangut thangkas and eleventh century Tibetan paintings. However, those supporting the Mongol/Yuan date also thought the painting design was closest to Tibetan paintings of the thirteenth/fourteenth century^4^. It was pointed out by Kossak^52^ that dating of Tibetan paintings was rather difficult as it relied mostly on connoisseurship rather than written words. The perils of dating by style alone and indeed by any method alone, even scientific methods, have been well illustrated by Ernst^35^. It was thought that Cave 465 included deities of various Tibetan tantric Buddhist sects making it difficult to assign it to a particular sect. This was used to support the early Tangut date because it was thought that in early Tangut period, Buddhism was not mature enough in the Tangut realm to have formed clear separation between the sects. Cave 465 is thought to be of Indo-Tibetan style, while all the Tangut cave temples in the Dunhuang region contained paintings that are more of a mixture of styles that included Chinese, Central Asian and Tibetan elements. This was thought to suggest that Cave 465 was constructed earlier than any of these caves, at the beginning of Tangut adoption of Tibetan Buddhist tradition and before they had the time to form their own style^3^. A more recent study of the iconography of Cave 465 has identified all the deities and suggested that the main deity of the cave is Cakrasaṃvara and that the cave temple most likely belonged to the Kagyü sect^54^. It also suggested briefly that Cave 465 appeared to be later than the other cave temples attributed to the Tangut period in the Dunhuang region but earlier than the thangkas excavated from Khara-Khoto based on the iconography and the content of the paintings. Meinert^55^ in her recent study also speculated that Emperor Renzong of the Tangut Empire may have been the patron of Cave 465, suggesting that Cave 465 was constructed in the last decades of the twelfth century.

Those arguing for a Mongol/Yuan date, mainly relied on archaeological evidence and the inscriptions^1,4,5^. Excavations conducted in the passageway between the middle hall and the neighbouring caves found fragments of documents in Chinese, Tibetan, Uyghur and Mongolian dated to the Mongol/Yuan period^5^. The earliest graffiti found in the middle hall (that is the current front hall) is dated to 1309 AD and the latest is 1373 AD. It was argued that the structure of the cave and its relations to the surrounding caves where excavations have been conducted, along with the graffiti and the style of the painting, suggested that the cave was constructed in the 2nd half of the thirteenth century^4^, that is the Mongol/Yuan period. However, the graffiti and the archaeological artefacts used in the arguments were exclusively from the middle hall and not the main hall.

In the current work, the material analysis showed that the pigment combination found on Cave 465 main hall paintings is most consistent with those of the Mongol/Yuan period, though Tangut period cannot be ruled out. The archaeological evidence also supports a Mongol/Yuan date. The printed Sanskrit text pasted below each of the Buddhas on the four ceilings for consecration of the paintings are found to be from after the late twelfth century based on palaeographic analysis. The prevalence of fourteenth century graffiti suggests that the cave temple was no longer looked after by the fourteenth century, which suggests that it must have been constructed before the fourteenth century. Taking into consideration all the evidences, the date of Cave 465 main hall wall paintings must be from the late twelfth to the thirteenth century.

## Methods

### Automated large-scale spectral imaging

The VIS/NIR version of the in-house developed PRISMS spectral imaging system consists of a Jenoptik CCD camera, a filter wheel with 10 filters from 400–880 nm (nine filters with bandwidth of 40 nm and central wavelength from 400 to 800 nm at 50 nm interval and a filter at 880 nm with a bandwidth of 70 nm) and a Meade ETX90 telescope^7^. It is capable of automatic imaging of large murals from a stand-off distance of 3–30 m at an angular resolution of 80 µrad giving both spectral and 3D topographic information (Fig. 2). The calibration of the images to obtain the spectral reflectance image cubes is automated and the detailed calibration procedure has been described in a previous paper^7^. This ten band reflectance spectral imaging system in the visible and near infrared range is a trade-off between maximising the capture of spectral features for material identification against cost in budget and time efficiency for large area surveys. Reflectance spectra of pigments and dyes tend to have smooth and broad spectral features which are adequately captured by the 10 band PRISMS system. Only in the case of a handful of pigments and dyes such as lac, madder or cobalt based pigments which have finer spectral features, is it necessary to employ high spectral resolution spectroscopy such as high resolution FORS or hyperspectral imaging systems^6^. Reflectance spectra extracted from PRISMS data cubes are used for clustering of similar spectra and for preliminary pigment identification.

### Automated generation of materials cluster map

The ‘kohonen’^23^ function from the built-in R stats package^56^ was used for the ‘Self-Organizing Map’ (SOM) clustering. Each image cube consists of over a million pixels and pixel-by-pixel clustering would be computationally slow. Therefore, we first reduce the dimensionality of the image cubes to a number of initial clusters by applying unsupervised SOM. The number of these initial clusters should be large enough to encompass all possible spectral groups within an image cube (e.g. 100 clusters; some of these clusters may belong to one larger cluster).

The automatic clustering of large number of spectral image cubes consists of three main steps (Supplementary Fig. S1): (1) unsupervised clustering of the initial spectral cube for the acquisition of the ‘Reference Spectral Database’, (2) automated clustering of large number of spectral image cubes, and (3) summary and production of the final cluster maps.

Unsupervised SOM is applied again on one initial spectral image cube after dimensionality reduction, clustering them into a smaller number of clusters (‘N-clusters’). This information is stored and constitutes the ‘Reference Spectral Database’. This number (N) can be an educated guess but does not need to be precise.

The sequential processing of large spectral imaging datasets is performed by applying supervised SOM, using the ‘Reference Spectral Database’ (Supplementary Fig. S2). The resultant clusters are then mapped on to the original full-sized image. Those pixels not matched to any of the existing clusters are grouped into a new cluster, the so-called ‘unclassified cluster’. Based on this mapping, the mean spectra and standard deviation (σ) of each cluster are determined. To confirm the pixel level clustering, the spectrum of each pixel of the original image is then compared again to the mean spectrum of each of the ‘N-clusters’ in the reference database. If it falls within the range of ± k·σ (k is usually around 2), the pixel is assigned to the corresponding cluster. All pixels that are not assigned to any of the reference ‘N-clusters’ are saved to the ‘unclassified cluster’. Finally, unsupervised SOM clustering is used to cluster the pixels in the ‘unclassified cluster’. The mean spectrum of each of the resulting clusters is compared with the mean spectra of each of the ‘N-clusters’. If it matches, the cluster is merged with the corresponding reference cluster, otherwise, it is added as a new cluster to the reference database. After processing each image cube, the reference database is updated and becomes ‘(N + w)-clusters’, where w is the number of new clusters added.

A final spectral comparison is performed between the clusters to confirm their spectral uniqueness. The ones with mean spectra matched within ± k·σ in all the spectral bands are merged. The final clusters are then mapped on to the set of original images, providing the final ‘cluster map’.

**Figure 7:**
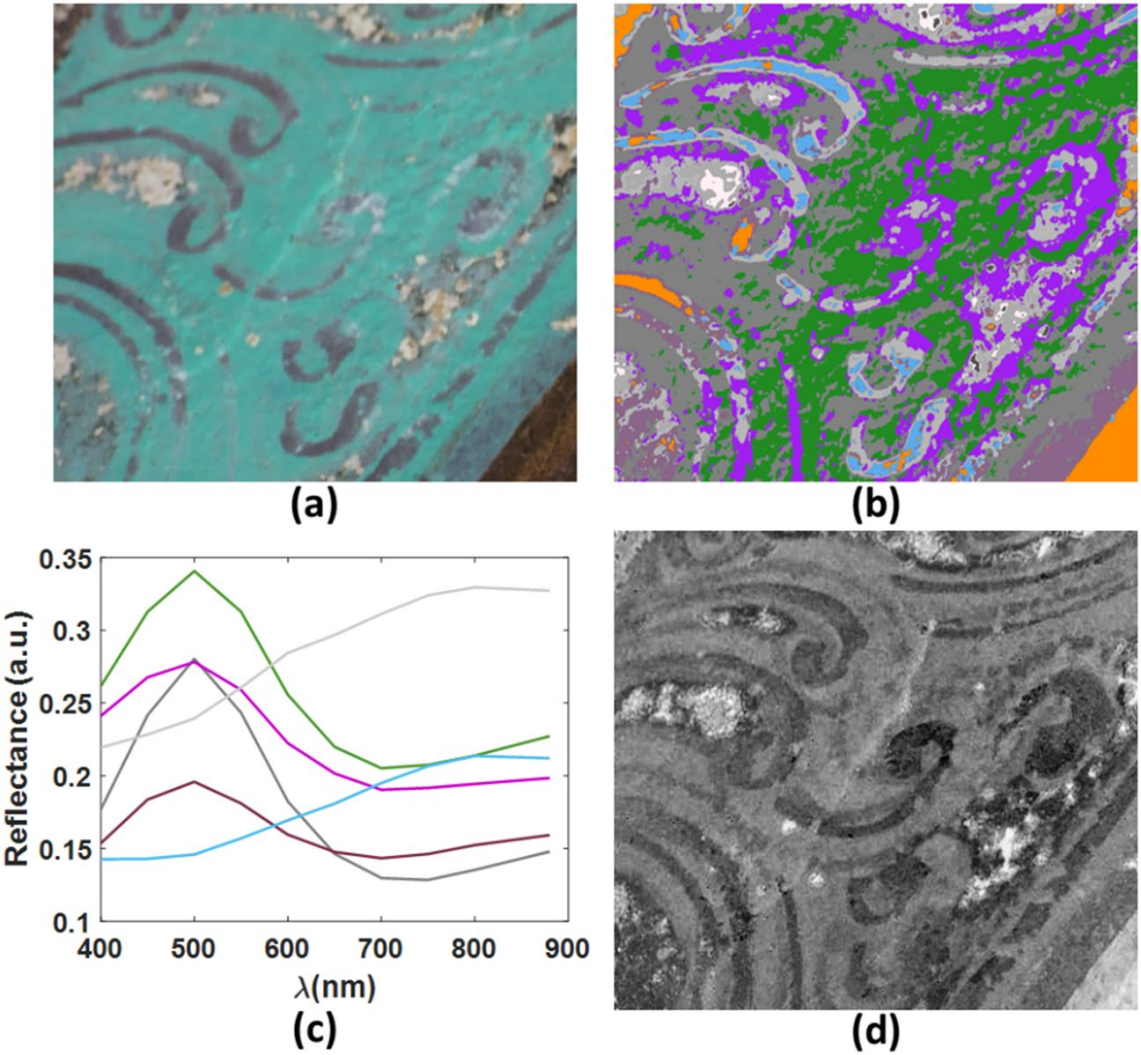
(**a**) Colour image derived from PRISMS spectral imaging data collected from a green area on the east ceiling of Cave 465, assuming CIE illuminant D65, 1931 2° standard observer; (**b**) corresponding cluster map of this area with a unique false colour assigned to each cluster; (**c**) mean spectra of the various clusters representing the various material mixtures, colour coded to match the colours of the cluster map in (**b**); (**d**) 3rd principal component of the spectral imaging data collected from this area showing the faded drawings (c.f. the cluster in (**b**) colour coded in purple).

The detailed cluster maps of the painting summarize the variations in the paint mixture, the thickness of the paint layers, particle size of the pigments, painting scheme and so on. Figure 7 shows the cluster map of one of the green areas. The green paint area is described by more than one cluster, but they all have the general spectral shape but different intensities which can correspond to different levels of dustiness, pigment concentration and particle sizes. All drawings share the same general spectral features at different intensities, indicating different levels of fadedness (Fig. 7c). Clustering revealed the presence of additional drawing patterns (Fig. 7b). Principle component analysis (PCA) of the spectral image cubes collected from this area confirmed the presence of faded drawings (Fig. 7d).

An example of the advantage of using both the spectral shape and the intensity of the reflectance in the clustering algorithm is demonstrated in the examination of the cloud pattern at the top of the east ceiling (Fig. 5). The analysis of the clusters that correspond to the red paint shows a spectral match with the red lead reference. The spectral comparison between the clusters of the red lead areas and the darker areas show a gradual reduction in the intensity of the spectra without a change in the spectral shape as it gets darker. This is due to the application of a thin wash of carbon ink on top of the red lead layer to achieve a darker shade.

The clustering algorithm enabled the distinction between areas with subtle visual differences. For example, the dark drawing materials used on the green atacamite (Fig. 7a) and those on the red lead areas (Fig. 5a) are different. The drawings on the red areas seem to have been painted using a carbon-based ink. On the other hand, the drawings on the green areas have the spectral feature of plattnerite (PbO_2_), a degradation product of red lead^39,40^, which suggests that the original drawings on the green area was red.

### Material identification using multimodal spectroscopic techniques

A range of complementary analytical techniques were used in the in situ, multimodal, non-invasive analysis of the wall paintings. High spectral resolution fibre optics reflectance spectroscopy (FORS) was performed using an ASD FieldSpec spectrometer, composed of three detectors that cover the spectral range from 350 to 2500 nm. The spectral resolution is 3 nm in the UV, visible and near infrared regime (350–1000 nm) and 10 nm in the short wave infrared (SWIR: 1000–2500 nm). Raman spectroscopy was performed using a mobile Horiba HE785 Raman spectrometer with laser excitation wavelength at 785 nm and laser power of 20 mW, coupled with a 50 × LWD (Long Working Distance) objective. The laser beam was focused to a spot approximately 30 µm in diameter. The spectral resolution is ~ 15 cm^−1^. Reference Raman spectra for pigments and dyes by Bell et al.^57^ and Burgio et al.^58^ were used for material identification. A portable Niton XL3t XRF Analyzer was used for X-ray fluorescence (XRF) spectroscopy. It consists of an Au anode, with maximum voltage at 50 kV and maximum current at 200 μA, enabling the detection of elements with atomic number Z > 14 in air.

Since similarities in reflectance spectra indicate similarities in material composition, clustering of areas at upper parts of walls and ceilings with areas on the ground level allows the extension of detailed pigment identification performed on the ground level to inaccessible parts of the wall painting. This detailed analysis was achieved following a multimodal approach with the combination of a series of non-invasive analytical techniques, from XRF and Raman to high spectral resolution FORS (see Supplementary Note 1). Kubelka–Munk model fit to PRISMS and FORS data was used for the identification of paint mixtures^6,59,60^.

### Automatic uncovering of ‘hidden’ writing and drawings

PCA and ICA were used to automatically process all spectral image datasets in order to reveal ‘hidden’ writings and drawings. For PCA, the ‘princomp’ function from the built-in R stats package was applied on the correlation matrix of the original spectral image cube and for ICA the ‘fastICA’ function was used.

## Supplementary information

Supplementary Information.

## Data Availability

The datasets generated during and/or analysed during the current study are available from the corresponding authors on reasonable request. The supplementary information shows all the multi-technique spectroscopy data used in this study.

## References

[1] Yang X, Duan W (1996). Dunhuang zangchuan mijiao yishu de zhengui yicun – mogaoku di 465 ku fu yulinku di 4 ku de neirong yu xingshi. Dunhuang Grotto Arts: Mogao Cave 465.

[2] Jin W, Duan W, Zhao M, Fan J, Liang W (1990). Dunhuang kukan mingshu kaobu. Dunhuang Shiku Yanjiu Guoji Taolunhui Wenji: Shiku Kaogubian.

[3] Xie J (2003). Mogaoku di 465 ku bihua hui yu xixia kao. Chin. Tibetol..

[4] Su B (1996). Zangchuan Fojiao Siyuan Kaogu.

[5] Peng J, Wang J (2004). Northern Grottoes of Mogaoku Dunhuang.

[6] Liang H (2012). Advances in multispectral and hyperspectral imaging for archaeology and art conservation. Appl. Phys. A.

[7] Liang H (2014). Remote spectral imaging with simultaneous extraction of 3D topography for historical wall paintings. ISPRS J. Photogr. Remote Sens..

[8] Koonsanit K, Jaruskulchai C, Eiumnoh A (2012). Band selection for dimension reduction in hyper spectral image using integrated information gain and principal components analysis technique. Int. J. Mach. Learn. Comput..

[9] Tsai F, Lin EK, Yoshino K (2007). Spectrally segmented principal component analysis of hyperspectral imagery for mapping invasive plant species. Int. J. Remote Sens..

[10] Wartenberg D (1985). Multivariate spatial correlation: A method for exploratory geographical analysis. Geogr. Anal..

[11] Naim A, Ratnatunga KU, Griffiths RE (1997). Galaxy morphology without classification: Self-organizing Maps. Astrophys. J. Suppl. Ser..

[12] Lu G, Fei B (2014). Medical hyperspectral imaging: A review. J. Biomed. Opt..

[13] Baronti S, Casini A, Lotti F, Porcinai S (1998). Multispectral imaging system for the mapping of pigments in works of art by use of principal component analysis. Appl. Opt..

[14] Striova J (2018). Spectral imaging and archival data in analysing Madonna of the rabbit paintings by Manet and Titian. Angew. Chem..

[15] Delaney JK (2014). Use of imaging spectroscopy, fibre optic reflectance spectroscopy, and X-ray fluorescence to map and identify pigments in illuminated manuscripts. Stud. Conserv..

[16] Mounier A, Daniel F (2015). Hyperspectral imaging for the study of two thirteenth-century Italian miniatures from the Marcadé collection, Treasury of the Saint-Andre Cathedral in Bordeaux, France. Stud. Conserv..

[17] Ricciardi P (2012). Near infrared reflectance imaging spectroscopy to map paint binders in situ on illuminated manuscripts. Angew. Chem.

[18] Delaney JK (2016). Use of near infrared reflectance imaging spectroscopy to map wool and silk fibres in historic tapestries. Anal. Methods.

[19] Boardman JW, Kruse FA, Green RO (1995). Mapping target signatures via partial unmixing of AVIRIS data. Summ. Fifth Annu. JPL Airborne Earth Sci. Workshop.

[20] Salerno E (2014). Analysis of multispectral images in cultural heritage and archaeology. JALS.

[21] Kohonen T (1982). Self-organized formation of topologically correct feature maps. Neural Netw..

[22] Kohonen T (2013). Essentials of the self-organizing map. Biol. Cybern..

[23] Wehrens R, Buydens LMC (2017). Self- and super-organizing maps in R: The Kohonen package. J. Stat. Softw..

[24] Kogou, S. Investigation of the complementary use of non-invasive techniques for the holistic analysis of paintings and automatic analysis of large scale spectral imaging data. *PhD Thesis* https://irep.ntu.ac.uk/id/eprint/32752/1/Sotiria%20Kogou%202018.pdf (2017).

[25] Attas M (2003). Near-infrared spectroscopic imaging in art conservation: Investigation of drawing constituents. J. Cult. Herit..

[26] Tonazzini A, Bedini L, Salerno E (2004). Independent component analysis for document restoration. IJDAR.

[27] Salerno E, Tonazzini A, Bedini L (2007). Digital image analysis to enhance underwritten text in the Archimedes palimpsest. IJDAR.

[28] Alexopoulou A, Kaminari A, Panagopoulos A, Pöhlmann E (2013). Multispectral documentation and image processing analysis of the papyrus of tomb II at Daphne Greece. J. Archaeol. Sci..

[29] Singer JC, O’Neill JP (1998). The cultural roots of early central Tibetan painting. Sacred Visions: Early Paintings from Central Tibet.

[30] Dalton JP, van Schaik S (2006). Tibetan Tantric Manuscripts from Dunhuang: A Descriptive Catalogue of the Stein Collection at the British Library.

[31] Samosyuk KF, Piotrovsky M (1999). Empire of the Silk Road, Buddhist Art from Khara Khoto X–XIIIth Century.

[32] Dunnell R (1992). The Hsia origins of the Yüan institution of imperial preceptor. Asia Major Third Ser..

[33] Lei Y, Wang S (2014). Material analysis of the wall paintings in Xialu temple Tibet Autonomous Region China. Stud. Conserv..

[34] Bruce-Gardner R, O’Neill JP (1998). Sacred Visions: Early Paintings from Central Tibet.

[35] Ernst RR (2001). Arts and sciences: A personal perspective of Tibetan painting. Chimia.

[36] Dunhuang Research Academy (1982). Dunhuang Mogaoku Neirong Zonglu.

[37] Dunhuang Research Academy (1996). Dunhuang Shiku Neirong Zonglu.

[38] Russell-Smith L (2005). Uyghur Patronage in Dunhuang: Regional Art Centres on the Northern Silk Road in the Tenth and Eleventh Centuries.

[39] Miguel C, Claro A, Gonçalves A, Muralha V, Melo M (2009). A study on red lead degradation in a medieval manuscript Lorvão Apocalypse (1189). J. Raman Spectrosc..

[40] Zhao Y (2016). Red lead degradation: Monitoring of colour change over time. New J. Chem..

[41] Xu W, Zhou G, Li Y (1983). Mogaoku bihua, caisu wuji yanliao de X shexian poxi baogao. Dunhuang Res..

[42] Guo H, Duan X (1995). Dongqianfodong bihua yanliao secai guilü ji bihua binghai zhili de yanjiu. Dunhuang Res..

[43] Duan X, Guo H, Fu W (1991). Mogaoku di 3 ku baozhenzhuang binghai de yanjiu – wenshidu guance ke zhizuo cailiao de fenxi. Dunhuang Res..

[44] Shi J (2009). Xixia huangshi he dunhuang mogaoku chuyi. Xixia Stud..

[45] Liu Y (1998). Dunhuang Xixia dongku fenqi zaiyi. Dunhuang Res..

[46] Boucher D (1991). The Pratityasamutpadagatha and its role in the medieval cult of the relics. J. Int. Assoc. Buddh. Stud..

[47] Salomon RG (1998). Indian Epigraphy: A Guide to the Study of Inscriptions in the Indo-Aryan Language.

[48] Ye S, Xie J, Xiong W, Miao Y, Lai T, Linrothe R, Ye S (2014). Feilaifeng shike fanwen tuoluoni de lanza ziti. Jiangnan Zangchuan Fojiao Yishu – Hangzhou Feilaifeng Shike Zaoxiang Yanjiu.

[49] Zhang B, Peng J, Wang J (2004). Dunhuang Sanskrit dharani. Northern Grottoes of Mogaoku Dunhuang.

[50] Singh AK (1990). Development of Nagari Script.

[51] Xie J (2000). Guanyu dunhuang di 465 ku duandai de jige wenti (xu). China Tibetol..

[52] Kossak SM, O’Neill SM (1998). The development of style in early central Tibetan painting. Sacred Visions: Early Paintings from Central Tibet.

[53] Xie J (2000). Guanyu dunhuang di 465 ku duandai de jige wenti. China Tibetol..

[54] Ruan L (2013). Mogaoku di 465 ku mantuoluo zaikao. Palace Museum J..

[55] Meinert C, Meinert C, Sørensen HH (2020). Creation of Tantric sacred spaces in Eastern Central Asia. Buddhism in Central Asia I: Patronage, Legitimation, Sacred Space, and Pilgrimage.

[56] R Development Core Team. R: a language and environment for statistical computing. https://www.R-project.org/, (2016).

[57] Bell I, Clark R, Gibbs P (1997). Raman spectroscopic library of natural and synthetic pigments (pre- ~1850 AD). Spectrochim. Acta Part A.

[58] Burgio L, Clear R (2001). Library of FT-Raman spectra of pigments, minerals, pigment media and varnishes, and supplement to existing library of Raman spectra of pigments with visible excitation. Spectrochim. Acta Part A.

[59] Liang, H., Keita, K., Peric, B., Vajzovic, T. Pigment identification with optical coherence tomography and multispectral imaging in *Proceedings of the 2nd International Topical Meeting on Optical Sensing and Artificial Vision 2008* 12–15 (ITMO State University, St Petersburg, 2008).

[60] Kogou S (2015). A holistic multimodal approach to the non-invasive analysis of watercolour paintings. Appl. Phys. A.

