# Supplementary material for: From remote sensing and machine learning to the history of the Silk Road: large scale material identification on wall paintings
Source: Sci Rep. 2020 Nov 9;10:19312. doi: 10.1038/s41598-020-76457-9 (PMC7652859; doi:10.1038/s41598-020-76457-9)
Supplement: Supplementary file 1 — Supplementary Information. [file 41598_2020_76457_MOESM1_ESM.pdf]

From remote sensing and machine learning to the history of the Silk Road: large scale material identification on wall paintings

Sotiria Kogou[a], Golnaz Shahtahmassebi[a], Andrei Lucian[a], Haida Liang[a*], Biwen Shui[b], Wenyuan Zhang[b], Bomin Su[b*], Sam van Schaik[c]

[a] School of Science & Technology, Nottingham Trent University, Nottingham NG11 8NS, UK
[b] Dunhuang Research Academy, Gansu Province, China
[c] The British Library, 96 Euston Road, London NW1 2DB, UK

*Corresponding authors: Haida Liang, Bomin Su
Email: Haida.Liang@ntu.ac.uk; Suboming@hotmail.com

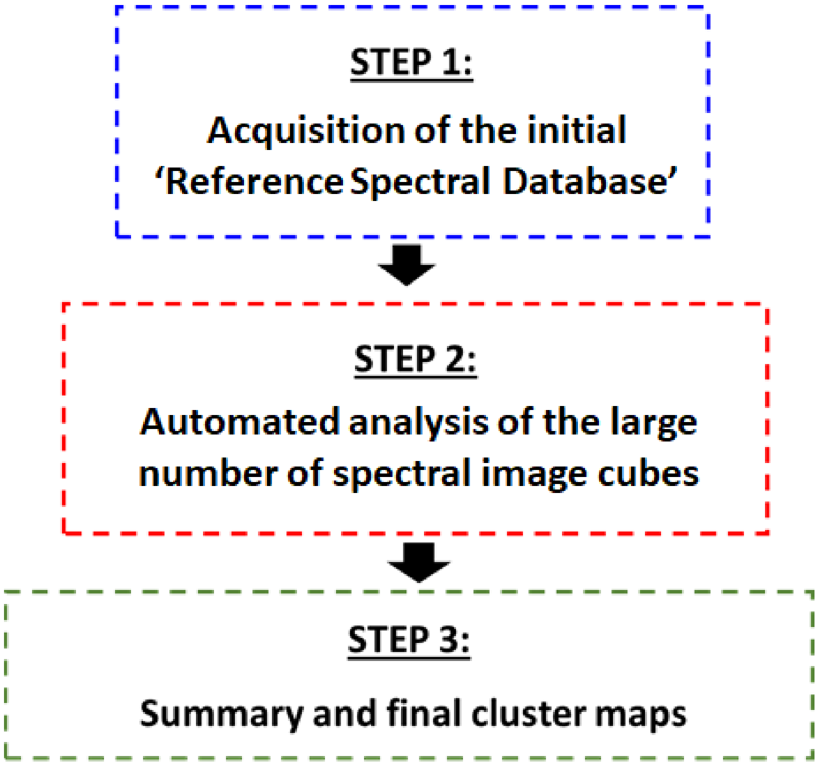

Supplementary Figure S1. An overview of the algorithm for automatic clustering of large number of spectral image cubes.

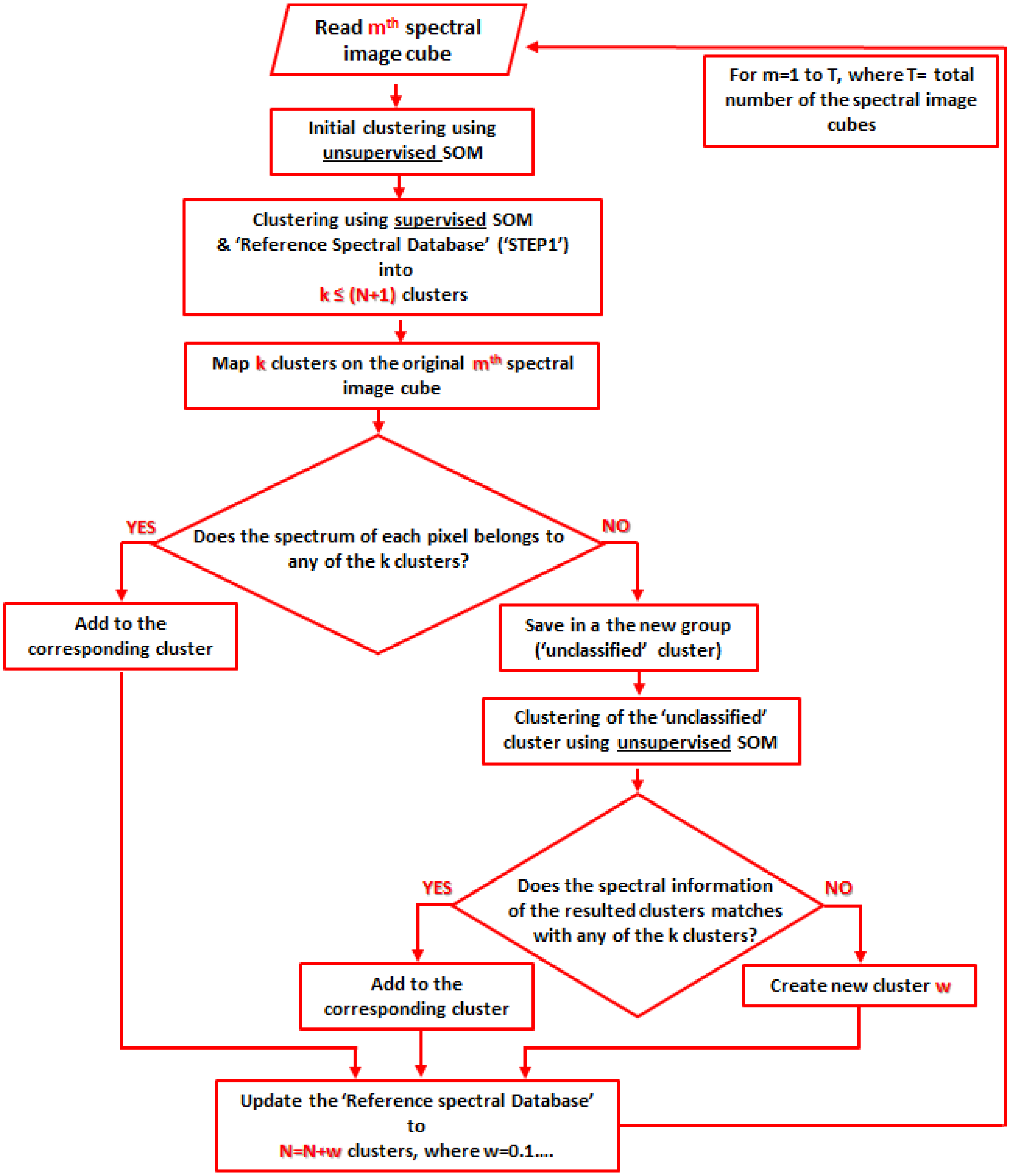

Supplementary Figure S2. Detailed description of 'Step 2' in Fig. S1 of the clustering algorithm for automated processing of large number of spectral image cubes.

Supplementary Table S1. Pigment composition from non-invasive analysis of Tibetan period Mogao paintings

Colour	Cave 159<br>*Spectral imaging, XRF, FORS*	Cave 386<br>*Spectral imaging, XRF*
**Red**	red ochre<br>cinnabar<br>arsenic pigment	red ochre<br>red ochre + arsenic pigment
**Blue**	lapis lazuli<br>azurite + lapis lazuli	lapis lazuli
**Green**	atacamite<br>malachite	atacamite
**White**	talc	-
**Brown/Black**	red ochre<br>plattnerite*<br>cinnabar + red lead + arsenic pigment + plattnerite*	red ochre<br>plattnerite<br>plattnerite + cinnabar<br>arsenic pigment
**Yellow**	yellow ochre	-

*Degradation product of red lead (turns it from red to brown/black)

Supplementary Table S2. Pigment composition from non-invasive analysis of Tangut period Mogao paintings

Colour	Cave 159<br>*Spectral imaging, XRF, FORS*	Cave 65<br>*Spectral imaging, XRF*	Cave 97<br>*XRF, FORS*
**Red**	red ochre	red ochre<br>red lead	red ochre<br>red ochre + arsenic pigment
**Blue**	-	lapis lazuli	azurite
**Green**	atacamite	atacamite	atacamite
**White**	-	talc	gypsum + dolomite
**Brown/Black**	plattnerite* + cinnabar	plattnerite* + cinnabar	red ochre + cinnabar + red lead

*Degradation product of red lead (turns it from red to brown/black)

Supplementary Table S3. Pigment composition from non-invasive analysis of Yuan period Mogao paintings

Colour	Cave 95<br>*Spectral imaging, Raman, XRF, FORS*
**Red**	cinnabar<br>red lead<br>red lead + cinnabar
**Blue**	azurite<br>indigo + azurite
**Green**	copper pigment
**White**	gypsum + dolomite
**Brown/Black**	cinnabar + plattnerite*
**Yellow**	orpiment<br>yellow ochre

*Degradation product of red lead (turns it from red to brown/black)

Supplementary Note 1:

Detailed analysis of the spectral clusters for pigment identification

Automatic clustering of the reflectance spectra collected by the PRISMS remote spectral imaging system mapped out the material variations, minimizing the need for detailed material identification to a manageable number of regions. In this section, the analysis of representative clusters of only unique pigment combinations is presented (e.g. clusters with the same pigment combination but different concentrations will not be discussed one by one). The analysis of the mean spectrum of each cluster, in combination with the multimodal complementary spectral analysis on representative areas of each cluster, provides a more comprehensive and accurate identification of the pigment composition of each cluster.

'Green 1' cluster. The spectral match between the 'Green 1' cluster of the ceiling data (Fig. 7 main paper) and some green areas on the ground level (Supplementary Fig. S3a) indicates similarities in their pigment composition. The NIR (or SWIR) part of the fibre optic reflectance spectroscopy (FORS) data (Supplementary Fig. S3b) collected from the green areas on the ground level has the characteristic absorption bands of atacamite ($Cu_2Cl(OH)_3$), indicating that this is the green pigment used. XRF spectra of these areas confirmed this identification by detecting Cu and Cl (Supplementary Fig. S3c), with Ca and Fe being part of the substrate.

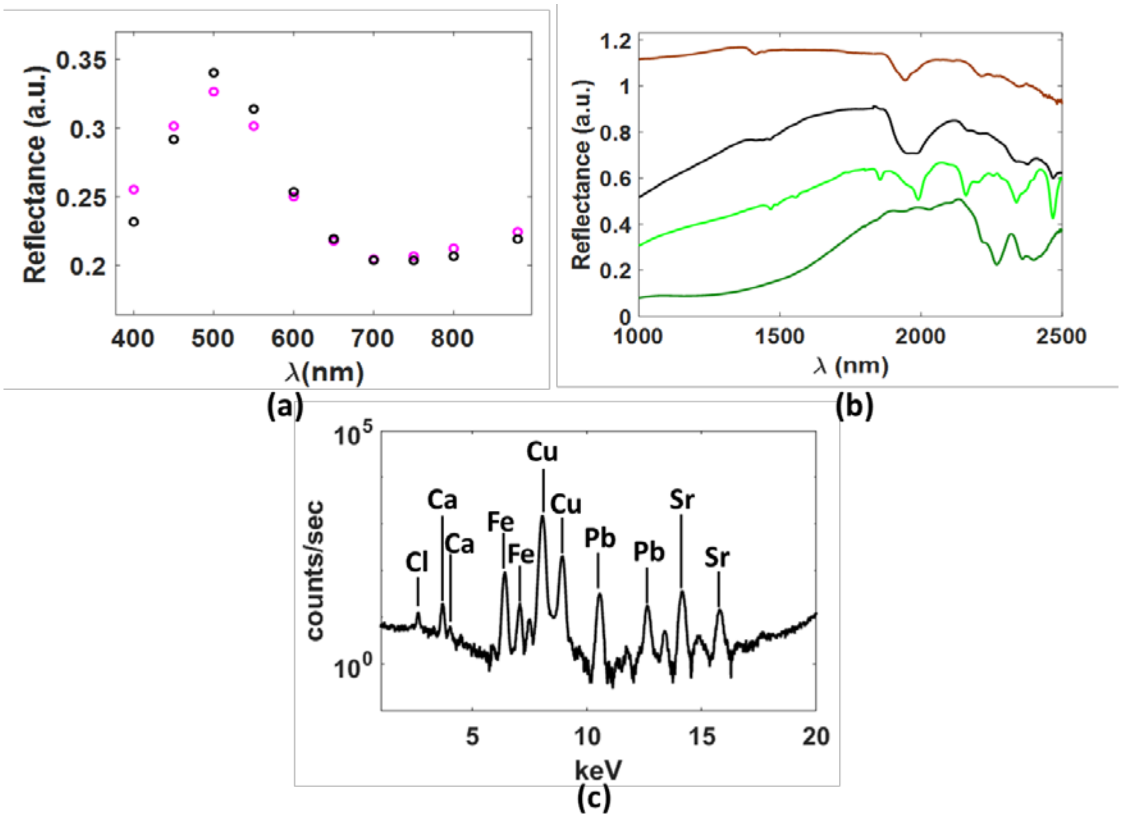

Supplementary Figure S3. (a) Spectral comparison between the two regions of 'Green 1' cluster: on the ceiling (magenta circles) and on the ground level (black circles); (b) the spectral comparison in the NIR regime between the green areas at the ground level (black curve), reference atacamite (bright green curve) and malachite (dark green curve) as well as the plaster layer (brown curve); (c) XRF spectrum of the green area on the ground level.

'Green 2' cluster. The Kubelka-Munk model gave a good fit to the 'Green 2' mean cluster spectrum using indigo (blue organic dye, $C_{16}H_{10}N_2O_2$) and the yellow pigment orpiment (As_2S_3) as references (Supplementary Fig. S4). However, reflectance spectroscopy alone is not enough to distinguish between the yellow pigments (except yellow ochre) [1]. A green area on the lower parts of the wall with a similar reflectance spectrum was subjected to XRF and Raman analysis that confirmed orpiment and indigo were used in these green areas.

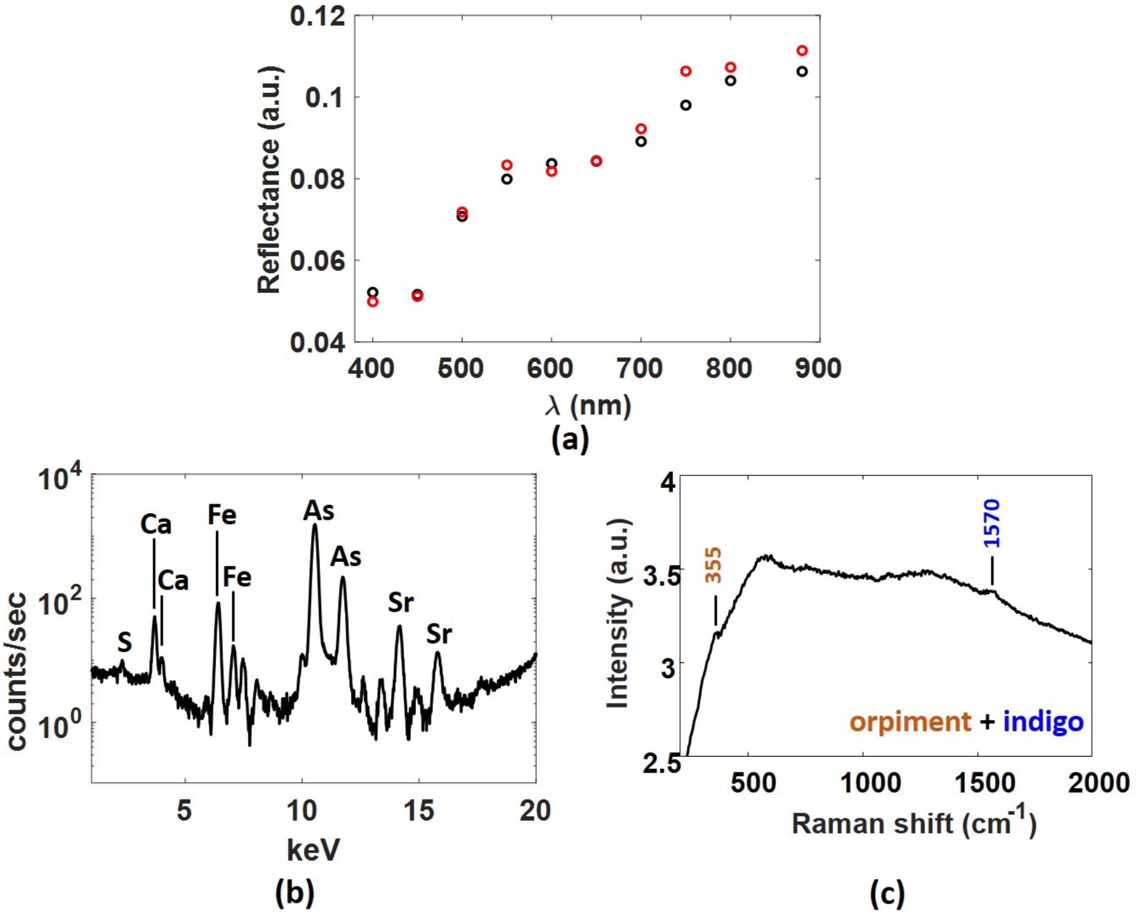

Supplementary Figure S4. (a) Kubelka-Munk model fit (red circles) on the 'Green 2' cluster of the east ceiling (black circles) using indigo and orpiment as references; (b) corresponding XRF spectrum; (c) corresponding Raman spectroscopy.

‘Blue 1’ cluster. Kubelka-Munk model using azurite ($2CuCO_3.Cu(OH)_2$) as reference gave a good fit to the spectrum of the ‘Blue 1’ cluster (Supplementary Fig. S5).

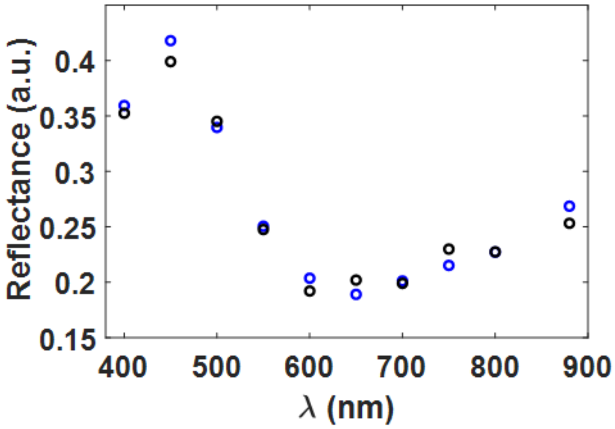

Supplementary Figure S5. The Kubelka-Munk model fit (black circles) to the ‘Blue 1’ cluster (blue circles) using azurite as reference.

‘Blue 2’ cluster. The ‘Blue 2’ cluster of the ceiling has a mean spectrum consistent with the FORS data collected from blue areas on the lower parts of the wall. Kubelka-Munk fit on the FORS data, in the VIS/NIR range, gave good match using a combination of azurite and indigo as references (Supplementary Fig. S6a). The extension of the reflectance spectra to the NIR regime showed that characteristic lines of azurite at 2284 nm and 2354 nm (Supplementary Fig. S6a inset), confirming its identification. Raman spectra collected from the same blue area (Supplementary Fig. S6b) also detected indigo and azurite lines.

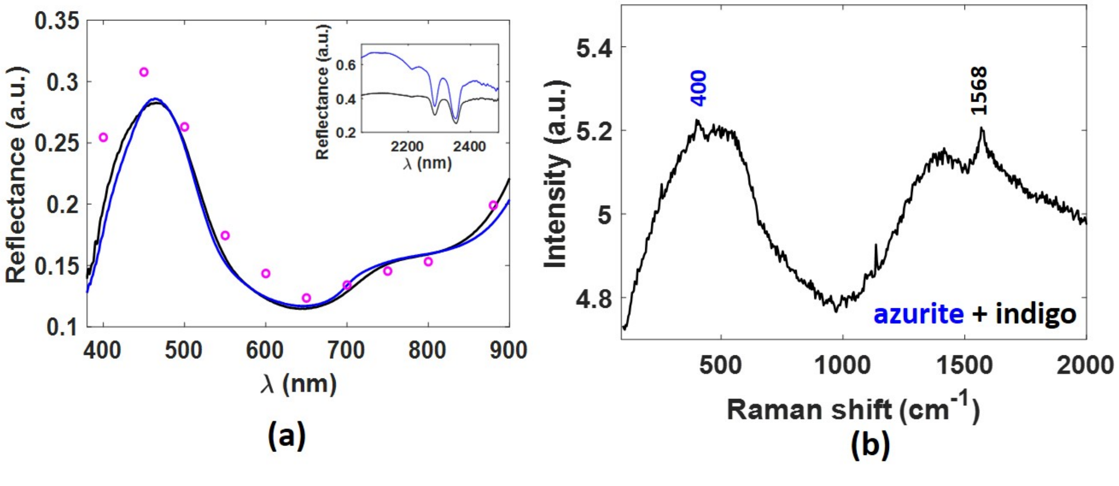

Supplementary Figure S6. (a) Spectral comparison between ‘Blue 2’ cluster of the ceiling (magenta circles) and FORS data collected from a blue area on a lower part of the mural (black curve). The Kubelka-Munk model (blue curve) gave the best fit on the FORS data (black curve) with a mixture of azurite with indigo; Inset: spectral comparison between the blue area (black curve) and the azurite reference (blue curve) in the NIR regime; (b) corresponding Raman spectrum.

'Blue 3' cluster. The Kubelka-Munk model fit to the 'Blue 3' cluster of the ceiling gave good match using indigo as reference (Supplementary Fig. S7a). Indigo was confirmed using Raman spectroscopy on the lower part of the wall with the same reflectance spectrum (Supplementary Fig. S7b).

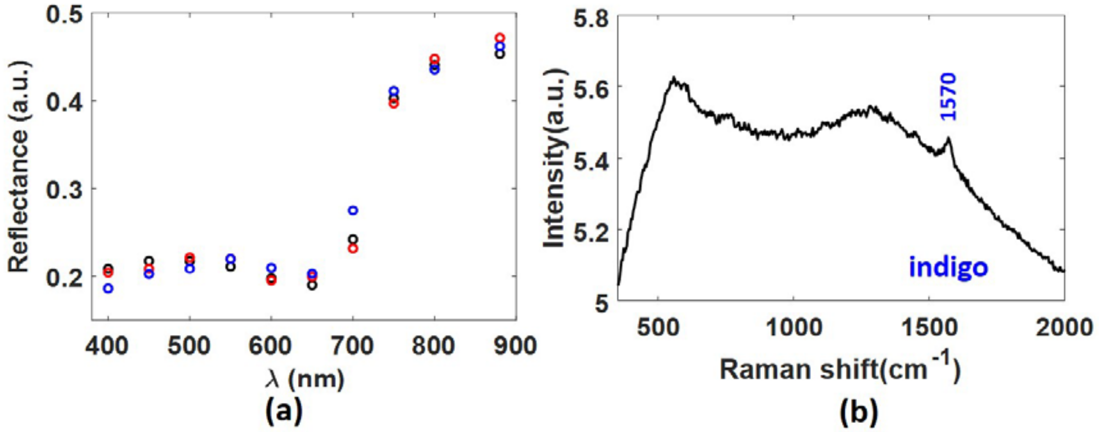

Supplementary Figure S7. (a) Comparison between the 'Blue 3' cluster of the ceiling (black circles) and PRISMS data collected from a grey/blueish area on the ground level (red circles), along with Kubelka-Munk model fit on the cluster using indigo as reference (blue circles). (b) corresponding Raman spectrum.

'Red 1' cluster. The Kubelka-Munk fit on the 'Red 1' cluster gave good fit using hematite (α-Fe_2O_3) as reference (Supplementary Fig. S8). Given that ochre has a distinctive feature in the visible regime, its identification is reliable without the need of further analysis.

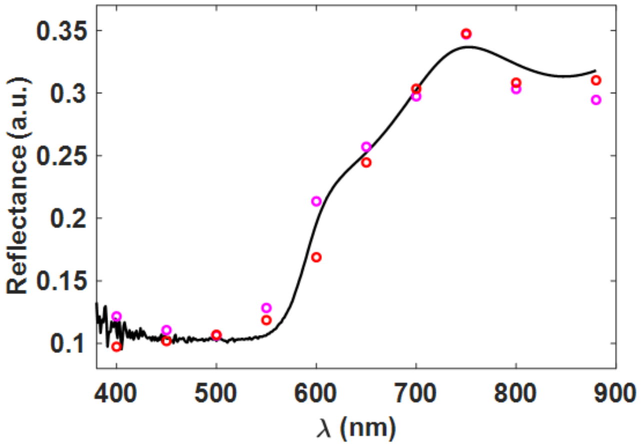

Supplementary Figure S8. Kubelka-Munk model gave the best fit (red circles) on the 'Red 1' cluster of the ceiling (magenta circles) using hematite as reference. The FORS spectrum of red ochre reference is also presented for comparison (black curve).

‘Red 2’ cluster. The spectral comparison between the ‘Red 2’ cluster of the east ceiling and red areas of the lower parts of the wall gave a good match (Supplementary Fig. S9a). XRF (Supplementary Fig. S9b) and Raman (Supplementary Fig. S9c) spectra clearly identified cinnabar (HgS).

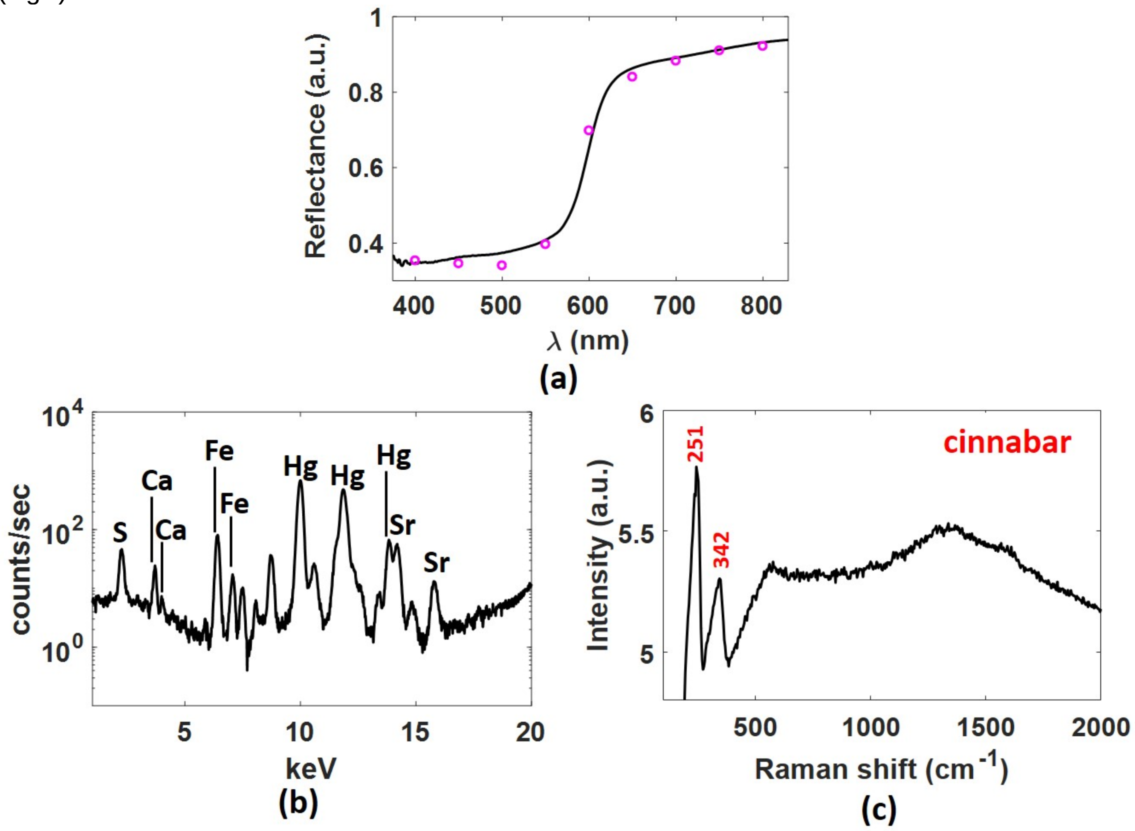

Supplementary Figure S9. (a) Comparison between the spectral feature of the ‘Red 2’ cluster of the east ceiling (magenta circles) and FORS data collected from a red area of the lower parts of the wall (black curve); corresponding (b) XRF spectrum and (c) Raman spectrum.

‘Red 3’ cluster. The spectral match between the ‘Red 3’ cluster of the ceiling and the FORS data collected from red areas on the lower part of the walls indicates similarities in their pigment composition (Supplementary Fig. S10a). XRF and Raman analysis confirmed red lead (Pb_3O_4). XRF found As suggesting likely presence of orpiment (As_2S_3) (Supplementary Fig. S10b, c).

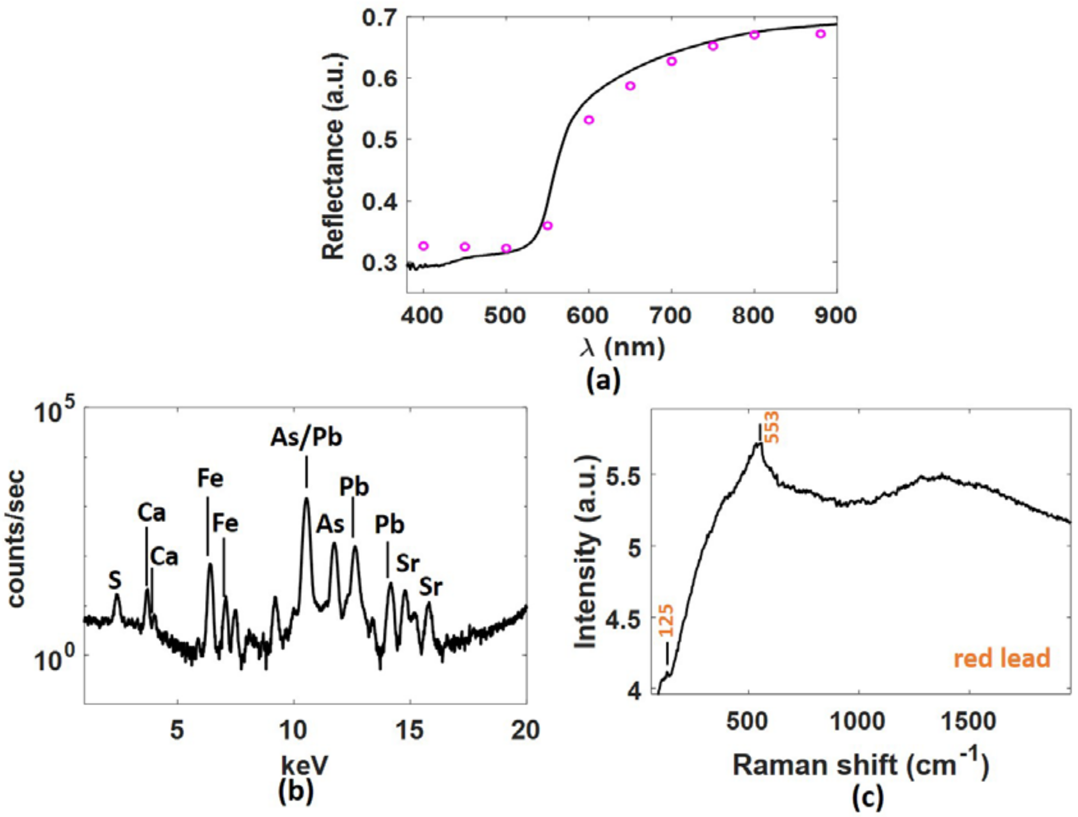

Supplementary Figure S10. (a) Spectral comparison of the ‘Red 3’ cluster of the east ceiling in Cave 465 (magenta circles) and the FORS data collected from an orange area at the ground level (black curve); corresponding (b) XRF spectrum and (c) Raman spectrum.

‘Red 5’ cluster. ‘Red 5’ cluster describes only areas of the ground level. Its multimodal analysis gave a combination of cinnabar, orpiment and red lead (Supplementary Fig. S11). The ‘Red 5’ area is painted on top of an indigo background that explains the weak indigo line in the Raman spectrum.

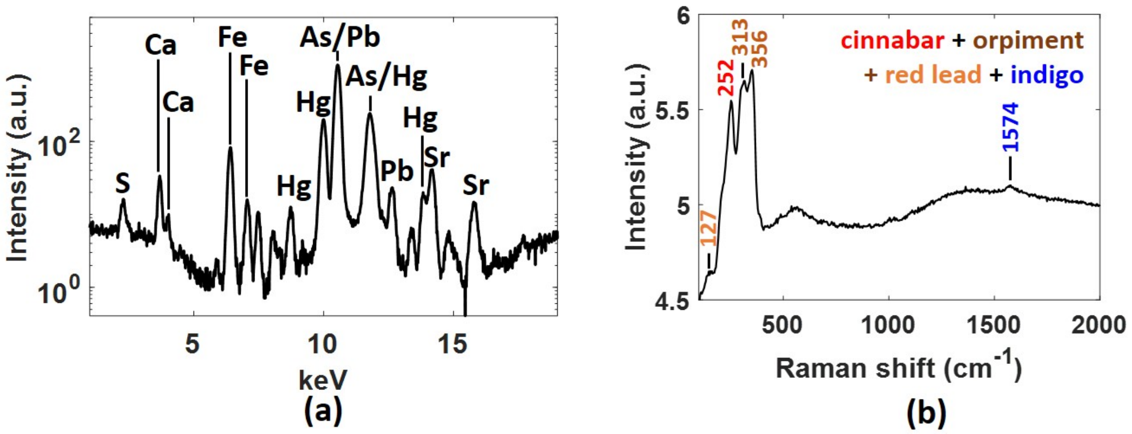

Supplementary Figure S11. (a) XRF spectrum of the ‘Red 5’ cluster and (b) the corresponding Raman spectrum.

‘Brown’ cluster. The mean reflectance spectrum of the ‘Brown’ cluster of the ceiling match the FORS data collected from a brown area on the ground level (Supplementary Fig. S12a), which showed the dominating presence of plattnerite (Supplementary Fig. S12b). The spectral feature of plattnerite (PbO_2) in reflectance spectrum with a peak around 750-800 nm is unique amongst the known pigments, making it easy to identify using reflectance spectroscopy. XRF spectroscopy (Supplementary Fig. S12c) found Pb as the major element and traces of Hg indicating the presence of cinnabar. Therefore, the original paint combination is assumed to consist of red lead and cinnabar, since plattnerite is a known degradation product of red lead.

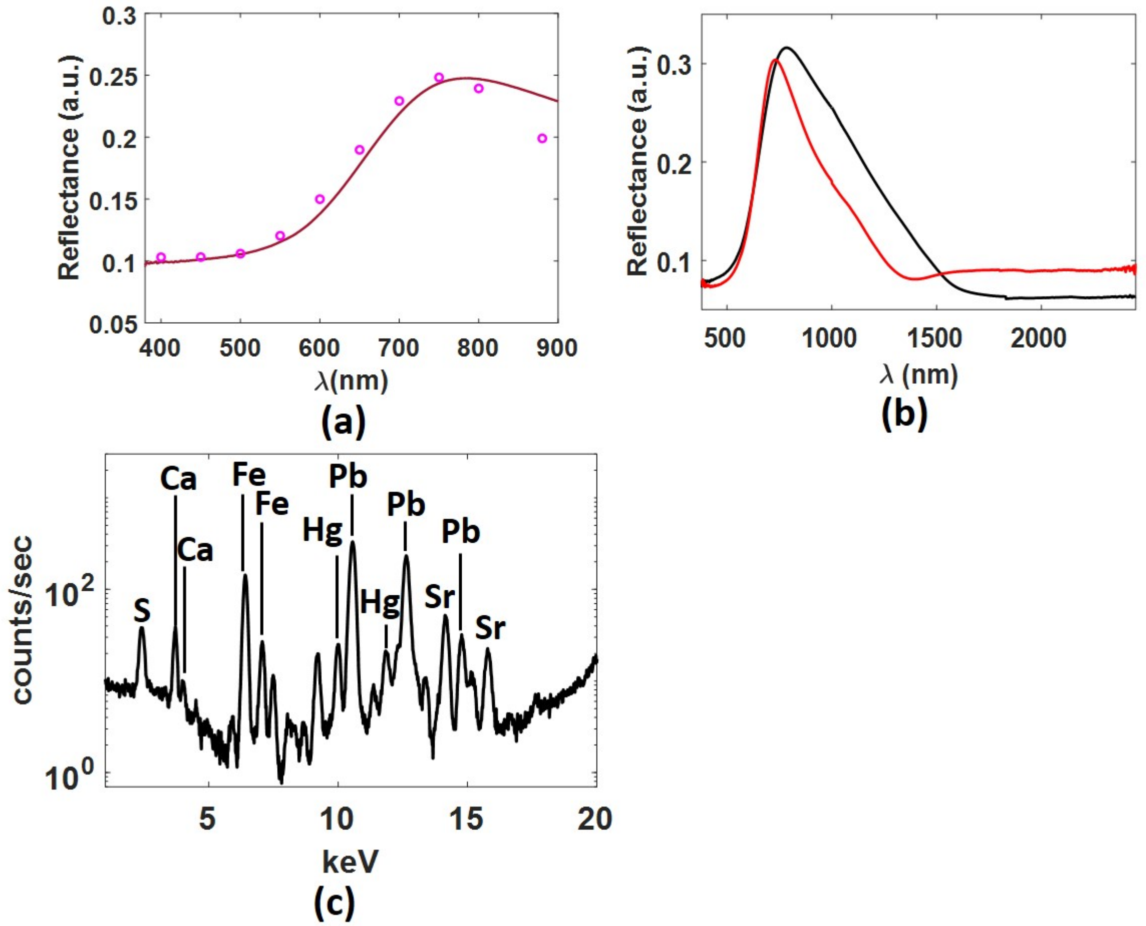

Supplementary Figure S12. (a) Spectral comparison between the ‘Brown’ cluster of the east ceiling (magenta circles) and the FORS spectrum collected from a brown area on the ground level (brown curve); (b) comparison of the FORS spectrum collected from a brown area on the ground level (black curve) to the spectrum of the artificially synthesized PbO_2 (red curve); c) XRF spectrum of the brown area on the ground level.

‘Lilac/Purple’ cluster. The ‘Lilac/Purple’ cluster that corresponds to the purple drawing on the green areas (Fig. 7 main paper) are spectrally matched with purple areas on the lower parts of the wall (Supplementary Fig. S13a) which shows the characteristic features of plattnerite. The XRF analysis (Supplementary Fig. S13b) on the purple areas on the ground level found Pb as the main element with trace Hg indicating cinnabar. The original paint must be a combination of cinnabar and red lead, since plattnerite is a degradation product of red lead.

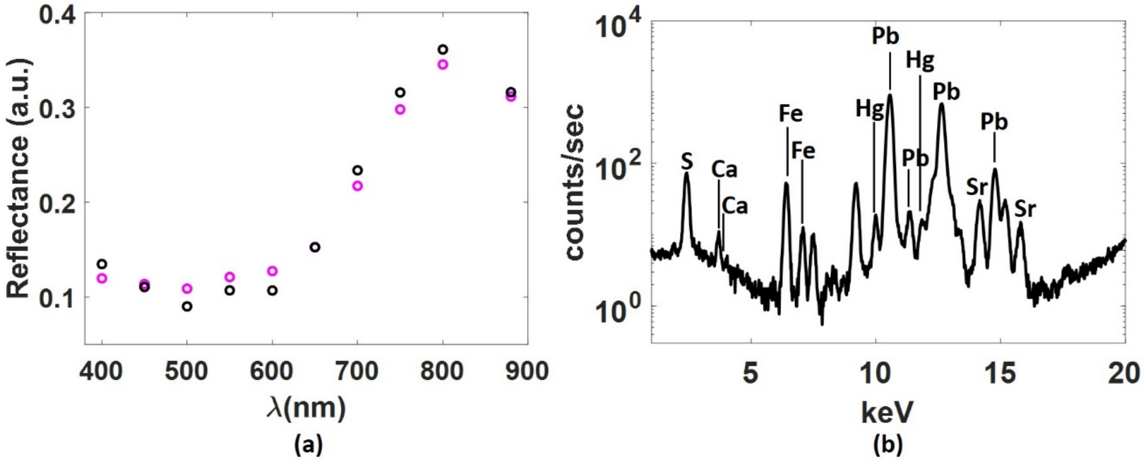

Supplementary Figure S13. (a) Spectral comparison between the ‘Lilac/Purple’ cluster that corresponds to the dark purple drawings (magenta circles) to PRISMS data on the lower parts of the wall that has been identified as containing plattnerite (black circles); (b) XRF measurement from the same area.

‘Yellow’ cluster. The mean spectrum of the ‘Yellow’ cluster of the yellow areas of the ceiling matches the PRISMS spectrum of a yellow decoration on the lower parts of the wall (Supplementary Fig. S14a). Raman analysis on the yellow areas on the ground level confirmed the identification of the yellow pigment as orpiment (Supplementary Fig. S14b).

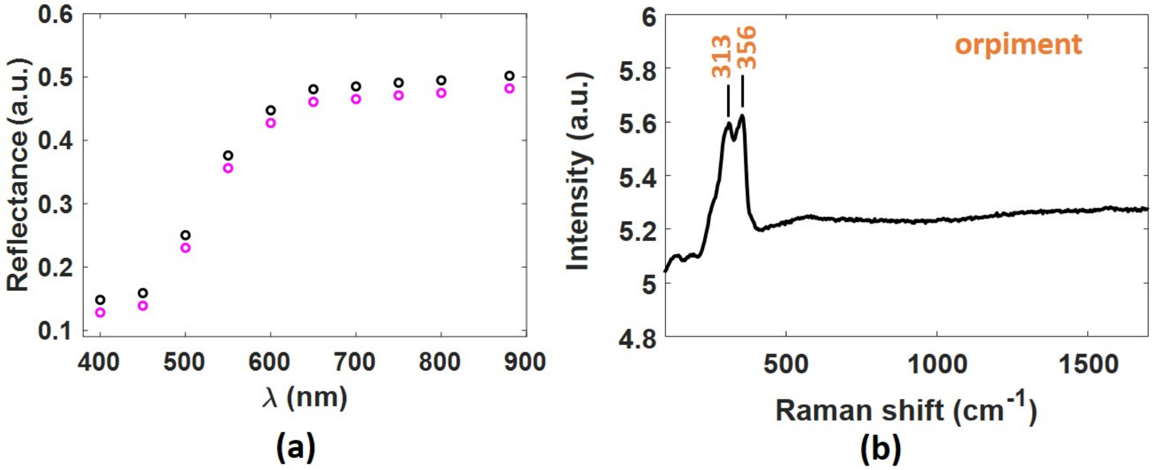

Supplementary Figure S14. (a) Spectral comparison between the ‘Yellow’ cluster of the ceiling (magenta circles) and the yellow decoration on the lower parts of the wall (black circles); (b) Raman spectrum of this area.

‘Black/blueish’ cluster. The ‘black/blueish’ cluster in areas on the ground level was found to be a combination of azurite and plattnerite (Supplementary Fig. S15) using XRF, FORS and Raman analysis. The original pigment combination must have been azurite and red lead, since plattnerite is a degradation product of red lead. The original colour would have been purple.

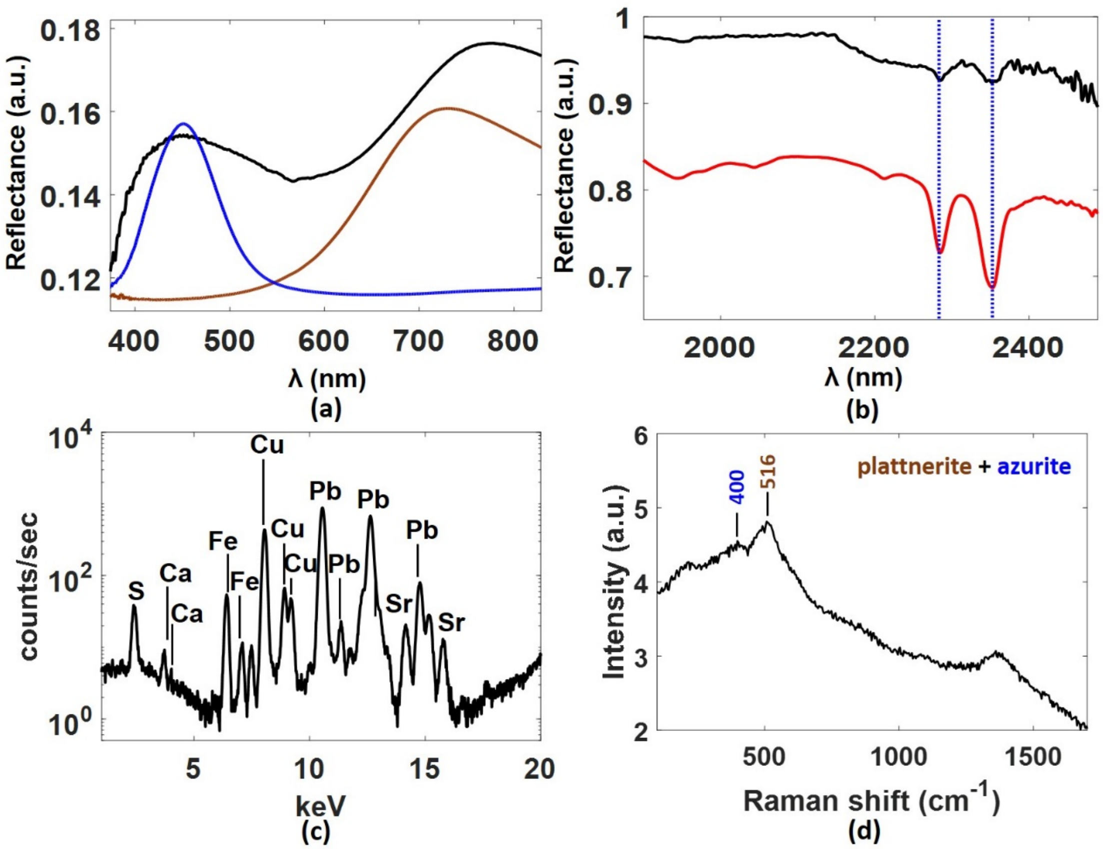

Supplementary Figure S15. (a) Visible part of the FORS spectrum collected from an area of the ‘Black/blueish’ cluster (black curve) compared with the reference spectra of azurite (blue curve) and plattnerite (brown curve); (b) Near-infrared part of the same spectrum (black curve) compared to the azurite reference (red curve), the vertical dashed lines indicate the position of the characteristic absorption lines; (c) XRF spectrum of the same area; (c) Raman spectrum collected from the same area.

Supplementary Note 2:

White pigments

Reflectance spectra in the NIR (or SWIR) regime enabled material identification of the white areas in different caves. Supplementary Figure S16 compares the spectra of the white areas and shows that caves 65 and 159 are very similar, while caves 95 and 465 are also very similar.

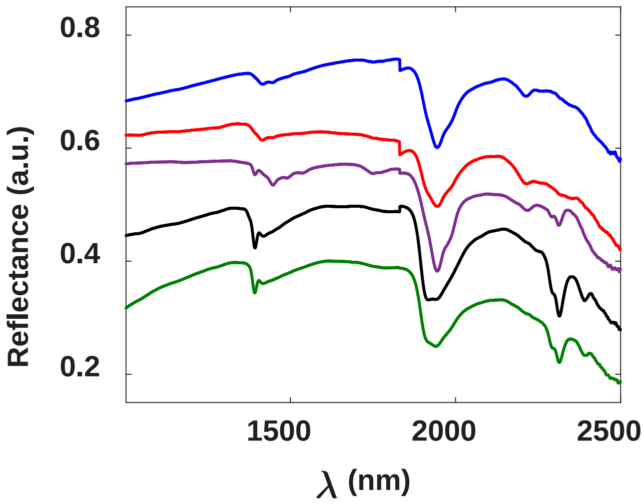

Supplementary Figure S16. Comparison between spectra collected from the white areas (top to bottom) of Cave 95 (blue curve), Cave 465 (red curve), Cave 97 (purple curve), Cave 65 (black curve) and of Tibetan period wall painting in Cave 159 (green curve). The spectra have been shifted vertically for clarity.

Supplementary Figure S17 shows that the white area in caves 159 and 65 are identified with the talc reference spectrum from the US Geological Survey (USGC) database.

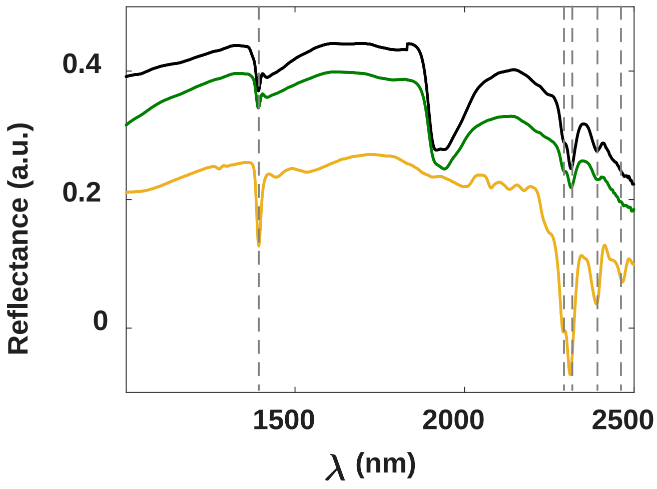

Supplementary Figure S17. Comparison (top to bottom) between the spectra collected from the white areas of Cave 65 (black curve) and of Tibetan period wall painting in Cave 159 (green curve), and the reference spectrum of talc from the USGS database (yellow curve). The vertical grey dashed lines are the absorption lines of the spectra that match with the reference.

Supplementary Figure S18 shows that white area in Caves 97, 95 and 465 are identified with a combination of gypsum and dolomite. Additional weak lines of calcite (e.g. at 2338 nm) in their spectra indicate either the use of a dolomite with a high calcite concentration or an addition of calcite. Cave 97 appears to have less calcite compared to the other two caves.

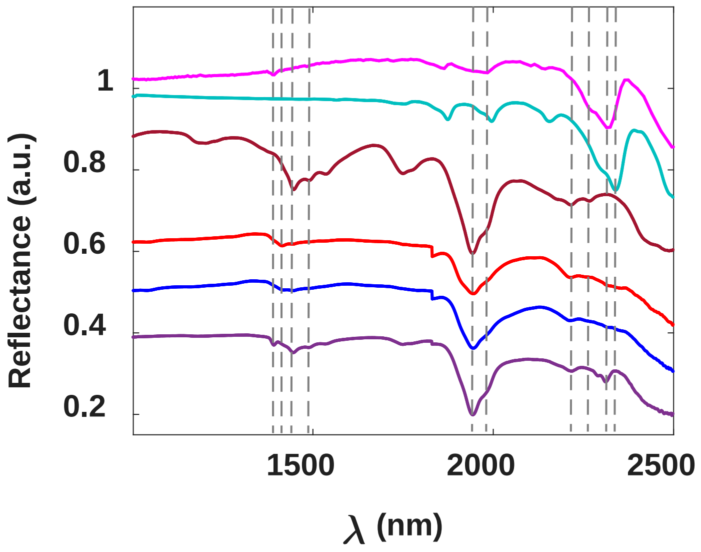

Supplementary Figure S18. Comparison (bottom to top) between the spectra collected from white areas of the Tangut Cave 97 (purple curve), the Yuan Cave 95 (blue curve), Cave 465 (red curve) and the reference spectrum of gypsum (brown curve), calcite (cyan curve) and dolomite (magenta curve) from the USGS database. The vertical grey dashed lines are the absorption lines that match with the reference.

Supplementary Note 3:

XRD and XRF analysis of samples from cave temple sites in the Dunhuang region

Dunhuang Academy has published a series of papers in the 1980s and 1990s on XRD and XRF analysis of physical samples taken from various cave temple sites in the Dunhuang region such as Mogao, Yulin and East Thousand Buddha Caves. Samples of representative colours were extracted from wall paintings dated to each different period in a cave. It was common that cave temples were renovated throughout their history. For example, at Mogao during the Tangut period, few new caves were built, but over 70 cave temples were repainted. Around 40 cave temples were attributed to the Tibetan period and only around 10 were constructed during the Yuan period. Note that samples extracted from polychrome sculptures were excluded here, partly because repainting of sculptures in the late Qing dynasty (1644-1911 CE) was fairly common and distinct differences in pigment compositions between wall paintings and sculptures were noted.

Supplementary Tables S4, S5 and S6 summarise the results from samples collected from those paintings attributed to the Tibetan period (781-847 AD), Tangut period (1036-1226 AD) and the Mongol/Yuan period (1227-1368 AD) respectively. Mogao caves 112, 197 and 231 were constructed during the Tibetan period, while Cave 188 was built in the High Tang period (705-780 AD) and renovated in the Tibetan period. Mogao Cave 245 was catalogued as a Tangut cave in the 1982 edition of the catalogue [2] but now considered to be painted in the Uyghur period, possibly in the 11th century, just before or at the beginning of the Tangut period [3]. Mogao caves 265 and 310 were built in the 5th/6th century and 6th/7th century respectively but renovated during the Tangut period. The Yulin caves 2, 3 and 29 are all from the late Tangut period but renovated in the Yuan period. Yulin Cave 4 was constructed in the Yuan period. Yulin Cave 29 is dated to 1193 AD based on an inscription. The East Thousand Buddha caves 2 and 5 were constructed during the Tangut period and Cave 7 was from the Yuan period. Details of the cave temples described in this section can be found in the catalogue published by Dunhuang Academy [3].

Supplementary Table S4. Pigment composition (XRD/XRF) of Tibetan period Dunhuang wall paintings

Cave	Red	Blue	Green	Brown/Black	White
**Mogao 188***	-	azurite	atacamite	plattnerite	talc+ calcite
**Mogao 197 ***	cinnabar<br>red ochre + red lead	azurite	atacamite	plattnerite	talc + calcite
**Mogao 231***	red ochre<br>cinnabar	lapis lazuli	malachite<br>atacamite	plattnerite	talc + calcite
**Mogao 112***	-	-	malachite<br>atacamite	-	mica<br>talc + calcite

* W. Xu, G. Zhou, Y. Li, Mogaoku Bihua, Caisu Wuji Yanliao de X Shexian Poxi Baogao, Dunhuang Research 1983(3), 187-197 (1983)

Supplementary Table S5. Pigment Composition (XRD/XRF) of Tangut period Dunhuang wall paintings

Cave	Red	Blue	Green	Brown/Black	White
**Mogao 265***	red ochre	-	atacamite	plattnerite + red lead	talc + calcite + gypsum
**Mogao 245***	red ochre	azurite lapis lazuli	atacamite	plattnerite + red lead	gypsum + anhydrite
**Mogao 310***	red ochre realgar + red lead	lapis lazuli	atacamite	plattnerite	gypsum + anhydrite
**Yulin 29†**	cinnabar	-	atacamite	-	talc
**East Thousand Buddha 2†**	cinnabar red lead	azurite azurite + lapis lazuli	atacamite	plattnerite + red lead $Pb_3Cl(AsO_3)_3$ azurite + plattnerite	calcite
**East Thousand Buddha 5†**	cinnabar red lead	-	atacamite	plattnerite + red lead	calcite gypsum

*W. Xu, G. Zhou, Y. Li, Mogaoku Bihua, Caisu Wuji Yanliao de X Shexian Poxi Baogao, Dunhuang Research 1983(3), 187-197 (1983)

†H. Guo, X. Duan, Dongqianfodong bihua yanliao secai guilü ji bihua binghai zhili de yanjiu, Dunhuang Research 1995(3), 59-73 (1995)

Supplementary Table S6. Pigment Composition (XRD/XRF) of Yuan period Dunhuang wall paintings

Cave	Red	Blue	Green	Yellow	Brown/Black	White
**Mogao 3***	cinnabar	azurite?	atacamite	As pigment	?	gypsum calcite
**Yulin 2†**	cinnabar red lead	azurite lapis lazuli	atacamite	-	$Pb_3Cl(AsO_3)_3$	calcite
**Yulin 3†**	red lead	azurite	atacamite	-	plattnerite	calcite
**Yulin 4†**	cinnabar red lead	azurite lapis lazuli	malachite	-	plattnerite $Pb_3Cl(AsO_3)_3$	calcite
**East Thousand Buddha 7†**	cinnabar red lead	azurite azurite + plattnerite red lead + plattnerite	atacamite	-	plattnerite red lead + plattnerite	calcite

*X. Duan, H. Guo, W. Fu, Mogaoku di 3 ku baozhenzhuang binghai de yanjiu – wenshidu guance ke zhizuo cailiao de fenxi, Dunhuang Research 1991(1), 103-121 (1991)

†H. Guo, X. Duan, Dongqianfodong bihua yanliao secai guilü ji bihua binghai zhili de yanjiu, Dunhuang Research 1995(3), 59-73 (1995)

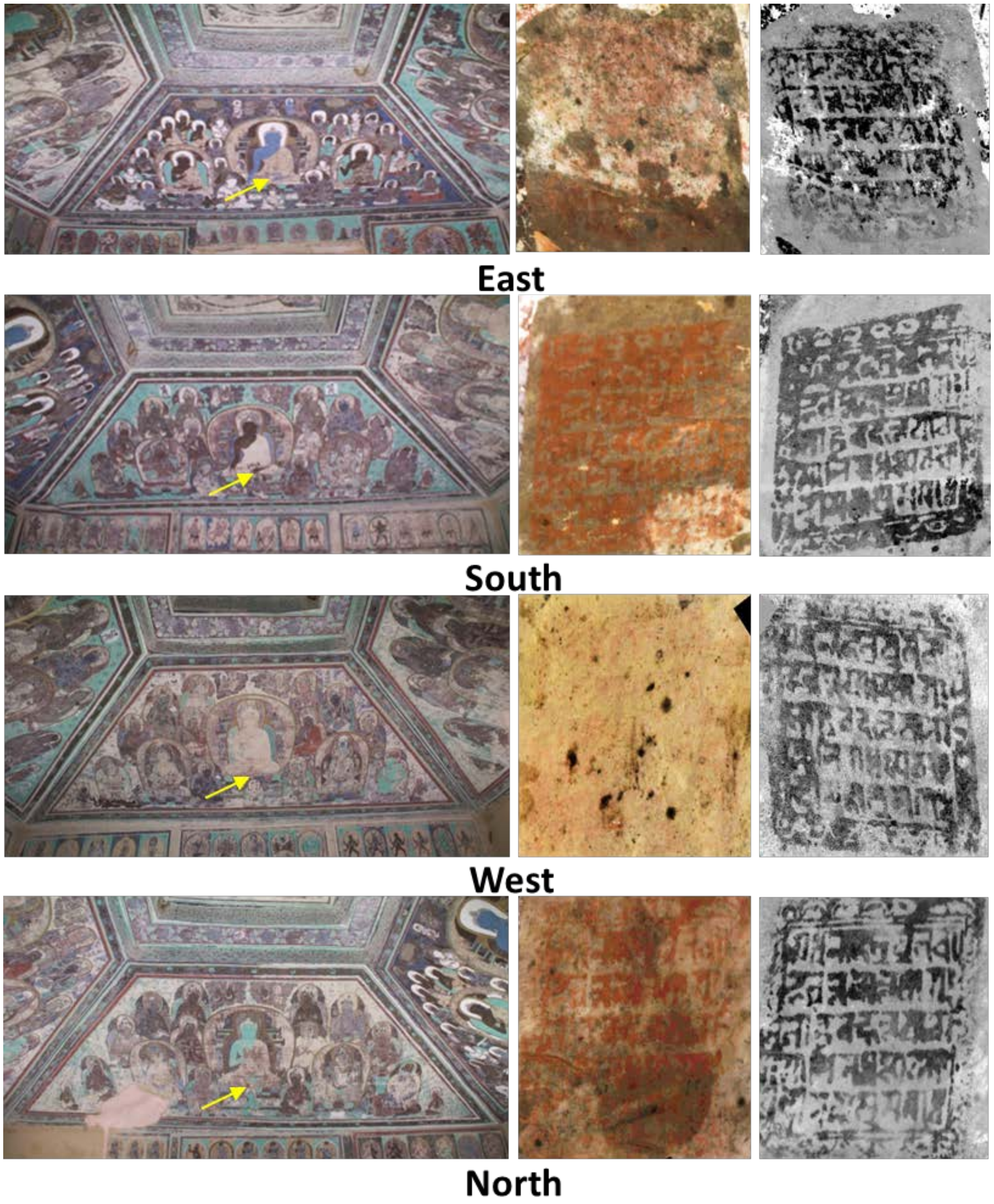

Supplementary Figure S19. The stamped text on paper sheets from the east, south, west and north ceilings. The position where the stamped texts were found on the ceilings are marked by yellow arrows. The colour image in the second column has been adjusted to show the best contrast for the writings. The images in the last column show the best processed images to enable reading of the text (note that the west one has been flipped horizontally so that the text is the right way around).

Supplementary Note 4:

Palaeographic analysis of Sanskrit text

The stamped Sanskrit text shown in Supplementary Fig. S19 are transcribed in Supplementary Table S7.

The usual phrase in Sanskrit is *ye dharmā hetuprabhavā hetuṃ teṣāṃ tathāgato hy avadat teṣāṃ ca yo nirodha evaṃ vādī mahāśramaṇaḥ*. There are a number of slight variations of the phrase [4] and differences in how the *sandhi* and *anusvara* are handled. Similar occurrence of ‘nta’ and ‘nte’ are found on the stamped sheets with the same Sanskrit phrase excavated from Cave 462 (462:2) and B168 (B168:48) [5] near Cave 465. In Central Asian Sanskrit, the vowels on their own are all variations of ‘a’ which in this case may explain the ‘e’ reading. The shape of the letters on the stamped text in Supplementary Fig. S19 are more consistent with the later stages of the development of the Nāgarī script in the 12th century when compared to inscriptions in India. In particular, the 1190s CE Jayachchandra inscription from Bodh-Gayā [6]. For example, the letters ‘yo’, ’ro’, ‘ṇa’, ‘ga’, ‘bha’ and ‘dha’ fits well with the shape of the letters from the Bodh-Gayā inscription but not any of the others in the reference set [6]. The letter ‘dha’ corresponds to emergence of the new form in the second half of the 12th century.

Supplementary Table S7. Transcribed letters from the printed text in Sanskrit.

ye	dha	rma	he	tu	pra	bha	vā
he	tu	nte	ṣā	nta	thā	ga	
to	ha	va	da	tte	sā	cā	
yo	ni	ro	dha	e	va	vā	
dī	ma	hā	śra	ma	ṇa	ḥ	

Supplementary References

1. Kogou, S. *et al.* A holistic multimodal approach to the non-invasive analysis of watercolour paintings, *Appl. Phys. A* **121(3)**, 999–1014 (2015).
2. Dunhuang Academy, Dunhuang Mogaoku Neirong Zonglu, (Wenwu Chubandshe, 1982).
3. Dunhuang Academy, Dunhuang Shiku Neirong Zonglu, (Wenwu Chubanshe, 1996).
4. von Hinüber, O., An Inscribed Avalokitesvara from the Hemis Monastery, Ladakh, *ARIRIAB XVIII*, 3-9 (2015).
5. Zhang, B. Northern Grottoes of Mogaoku Dunhuang (Wenwu Chubanshe, 2004).
6. Singh, A. K. Development of Nagari Script (Parimal Publications, 1990).
